# First carrot, then stick: how the adaptive hybridization of incentives promotes cooperation

**DOI:** 10.1098/rsif.2014.0935

**Published:** 2015-01-06

**Authors:** Xiaojie Chen, Tatsuya Sasaki, Åke Brännström, Ulf Dieckmann

**Affiliations:** 1 Evolution and Ecology Program, International Institute for Applied Systems Analysis (IIASA), Laxenburg 2361, Austria; 2 School of Mathematical Sciences, University of Electronic Science and Technology of China, Chengdu 611731, People's Republic of China; 3 Faculty of Mathematics, University of Vienna, Vienna 1090, Austria; 4 Department of Mathematics and Mathematical Statistics, Umeå University, Umeå 90187, Sweden

**Keywords:** punishment, reward, public good, evolutionary game, social design

## Abstract

Social institutions often use rewards and penalties to promote cooperation. Providing incentives tends to be costly, so it is important to find effective and efficient policies for the combined use of rewards and penalties. Most studies of cooperation, however, have addressed rewarding and punishing in isolation and have focused on peer-to-peer sanctioning as opposed to institutional sanctioning. Here, we demonstrate that an institutional sanctioning policy we call ‘first carrot, then stick’ is unexpectedly successful in promoting cooperation. The policy switches the incentive from rewarding to punishing when the frequency of cooperators exceeds a threshold. We find that this policy establishes and recovers full cooperation at lower cost and under a wider range of conditions than either rewards or penalties alone, in both well-mixed and spatial populations. In particular, the spatial dynamics of cooperation make it evident how punishment acts as a ‘booster stage’ that capitalizes on and amplifies the pro-social effects of rewarding. Together, our results show that the adaptive hybridization of incentives offers the ‘best of both worlds’ by combining the effectiveness of rewarding in establishing cooperation with the effectiveness of punishing in recovering it, thereby providing a surprisingly inexpensive and widely applicable method of promoting cooperation.

## 1. Introduction

Cooperation is desirable whenever groups of cooperating individuals can reap higher benefits than groups of individuals acting out of individual self-interest. Promoting cooperation can be difficult, however, because a single non-cooperating individual (‘defector’) in a group of cooperators often achieves a higher net benefit by free-riding on the others' contributions. An efficient policy for promoting cooperation needs to overcome two fundamental challenges: to ensure that cooperators can gain a foothold in a population of defectors and to protect a population of cooperators from exploitation by defectors once cooperation has been established.

Incentives can help overcome these challenges [1–3]. The promise of reward or the threat of punishment can induce cooperation among self-interested individuals who would otherwise prefer actions that undermine the public good. At first glance, there might seem to be little difference between a reward and a penalty: after all, cooperation is induced whenever the size of the incentive exceeds the pay-off difference between a cooperator and a defector, regardless of whether the incentive is positive or negative [4]. This equivalence ceases to hold, however, when one considers the costs of implementing an incentive scheme. Rewarding a large number of cooperators or penalizing a large number of defectors is either very costly or becomes ineffective when a limited budget for incentives is stretched out too far. Pamela Oliver exemplifies this with the problem of fundraising: ‘If only 5% of the population needs to contribute to an Arts Fund for it to be successful, they can be rewarded by having their names printed in a program: it would be silly and wasteful to try to punish the 95% who did not contribute’ [5, p. 125]. While the challenges of implementing positive and negative incentives are separately well known [2,3] and weight has traditionally been given to peer-to-peer punishment [6–8], no study to date has established how such incentives should best be combined at an institutional level to promote cooperation.

Here, we demonstrate how an institution implementing incentives can effectively establish and recover cooperation at low cost. Institutional sanctioning is widespread [1,4,9–22]; however, surprisingly few theoretical studies have thus far considered the effects of institutionalized incentives on the evolution of cooperation, and the few studies that exist have either considered rewarding and punishing in isolation [4,9,19] or did not consider how optional incentives change with the frequency of cooperators [10–12]. Indeed, sanctioning agents, such as officers and managers, often alter rewards and penalties as events unfold. We address this question in an established game-theoretical framework for studying the evolution of cooperation under institutionalized incentives [4,19]. By considering the strengths of positive and negative incentives as independent variables, we can encompass a range of hybrid incentive policies. In particular, by allowing the relative allocation of incentives to rewarding and punishing to vary with the frequency of cooperators, our framework includes hybrid incentive policies controlled by adaptive feedback from the population's current state.

## 2. Model and methods

### 2.1. Institutional incentives

We aim to determine the best way to allocate a budget available to an institution for promoting cooperation through positive and negative incentives. As criteria for assessing the performance of alternative sanctioning policies, we consider their effectiveness and efficiency in promoting cooperation. For measuring effectiveness, we assess the diversity of conditions for which full cooperation can be established or recovered with certainty, and for measuring efficiency, we determine the cumulative cost and total time required to convert a population of defectors to full cooperation or to recover full cooperation after the invasion of a single defector.

### 2.2. Public good games with dynamic incentives

Our model is based on the public good game for cooperation (C) and defection (D), widely recognized as the most suitable mathematical metaphor for studying cooperation in large groups [23–28]. We posit well-mixed populations of interacting individuals. From time to time, individuals randomly selected from the population form an *n*-player group with *n* ≥ 2. A cooperator invests a fixed amount *c* > 0 into a common pool, whereas a defector invests nothing. The total contribution to the pool is then multiplied by a public-benefit factor *r* > 1 and distributed equally among all *n* group members. The infamous ‘tragedy of the commons’ [29] arises when *r* < *n* and no incentives are applied, because single individuals can then improve their pay-offs by withholding their contributions.

The total budget for providing incentives is given by *nδ* per group, where *δ* > 0 is the average *per capita* incentive. This budget *nδ* is then divided into two parts based on a relative weight *w* with 0 ≤ *w* ≤ 1. The part *wnδ* is equally shared among the *n*_C_ cooperators in the group (see Chen *et al.* [17] for a similar application to the *n*-person volunteer's dilemma), who thus each obtain a reward *awnδ*/*n*_C_, while the remainder (1 − *w*)*nδ* is used for equally punishing the *n* − *n*_C_ defectors, who thus each have their pay-offs reduced by *b*(1 − *w*)*nδ*/(*n* − *n*_C_). The factors *a*,*b* > 0 are the respective leverages of rewarding and punishing, i.e. the factors by which a recipient's pay-off is increased or decreased relative to the cost of implementing the incentive. ‘Antisocial’ incentives, rewarding defectors or punishing cooperators [30], could in principle be considered, but as such incentives only reduce cooperation and promote defection, they are not studied here.

We account for feedback from the population's state by allowing the weight *w* to depend on the frequency of cooperators. Pure rewarding and pure punishing correspond to *w* = 1 or *w* = 0, respectively. Therefore, a cooperator and a defector obtain the pay-offs2.1
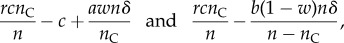
respectively.

### 2.3. Replicator dynamics

We assume replicator dynamics [31], which describe how the frequencies of different strategies change in infinitely large, well-mixed populations. Replicator dynamics are governed by a system of differential equations, 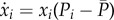, in which *x_i_*, *P_i_* and 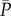 denote, respectively, the frequency of strategy *i*, the average pay-off for strategy *i*, and the average pay-off in the whole population (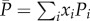, with 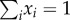). In the public good game studied here, we consider cooperation and defection with respective frequencies *x* and 1 − *x*. The replicator dynamics are therefore given by a single differential equation, 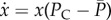. With 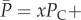 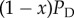, we obtain2.2
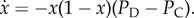
This differential equation has at least two equilibria: *x* = 0, at which all individuals defect, and *x* = 1, at which all individuals cooperate.

Our model extends the traditional public good game [23–28] by incorporating incentives. Specifically, letting *k* denote the number of cooperators among the *n* − 1 co-players in a group, the expected pay-offs for a defector and a cooperator are given by2.3a
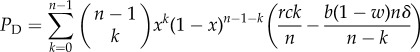
and2.3b
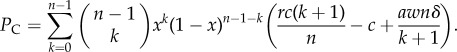
Without incentives, *δ* = 0, we have *P*_D_ − *P*_C_ = *c*(1 − *r*/*n*) = *F*, which is the defector's advantage in the public good game. The replicator dynamics in equation (2.2) thus lead to full defection, *x* = 0, when *r* < *n* and to full cooperation, *x* = 1, when *r* > *n*. According to equations (2.3), incentives *δ* > 0 modify the defector's advantage for 0 < *x* < 1 as follows:2.4a
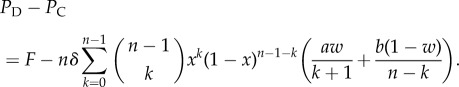
Using 
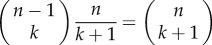
 and 
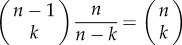
, this yields2.4b
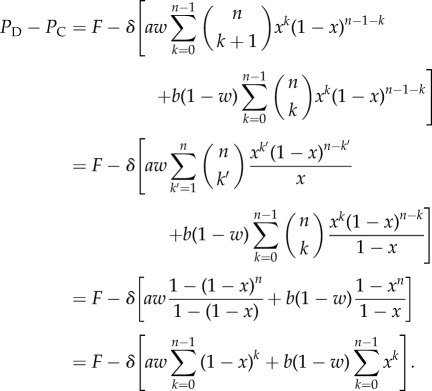
When rewarding or punishing are applied in isolation (*w* = 1 or *w* = 0), the defector's advantage simplifies to 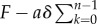 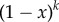 and 
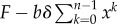
, respectively [4,19]. Thus, the defector's advantage is strictly increasing with the frequency *x* of cooperators for pure rewarding and strictly decreasing with the frequency *x* for pure punishing. In both the cases, there exists a unique interior equilibrium of the replicator dynamics if and only if the *per capita* incentive *δ* lies within an intermediate range, *δ*_−_ < *δ* < *δ*_+_, with2.5
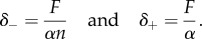
Here the value of *α* depends on the type of incentive being applied: *α* = *a* for rewarding and *α* = *b* for punishing. The unique interior equilibrium is globally asymptotically stable for pure rewarding and unstable for pure punishing. Therefore, when incentives are intermediate, *δ*_−_ < *δ* < *δ*_+_, the replicator dynamics for pure rewarding lead to a mixture of defectors and cooperators, while for pure punishing, they lead to bistability between full defection and full cooperation. By contrast, if incentives are very small, *δ* ≤ *δ*_−_, or very large, *δ* ≥ *δ*_+_, the replicator dynamics for pure rewarding and for pure punishing lead to full defection or full cooperation, respectively.

## 3. Results

We first demonstrate, in §3.1, that an institutional sanctioning policy we call ‘first carrot, then stick’, which switches from rewarding to punishing when the frequency of cooperators exceeds a threshold, minimizes the defector's advantage. Its effectiveness and efficiency are investigated in §3.2 and compared with those of pure rewarding and pure punishing. We extend our results to spatial populations in §3.3 and conclude by verifying, in §3.4, that our results are robust to other parameter combinations and other model variants.

### 3.1. ‘First carrot, then stick’ as an optimal sanctioning policy

By allowing the fraction *w* of the incentive budget that is allocated to rewarding, rather than to punishing, to change with the frequency of cooperators, *w* = *w*(*x*), we can represent a broad range of institutional sanctioning policies. Below we show that the ‘first carrot, then stick’ sanctioning policy is optimal in that it minimizes the defector's advantage *P*_D_ − *P*_C_; it thus maximizes the selection gradient 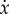 at any frequency *x* of cooperators. This means that the ‘first carrot, then stick’ sanctioning policy results in the highest level of cooperation for each parameter combination and that it consequently is the most effective institutional sanctioning policy.

To see that the ‘first carrot, then stick’ sanctioning policy minimizes the defector's advantage, we first write equation (2.4*b*) as3.1
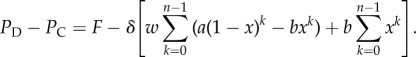
As this equation is linear with respect to the weight *w*, a value of *w* of either 0 or 1 is optimal depending on whether the sum 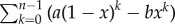 is positive or negative, respectively. (In the degenerate case when this sum equals zero, any value of *w* will be optimal.) One can show that this sum is a decreasing function of *x* with exactly one root 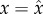 satisfying 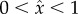, about which the sum changes sign from positive to negative as *x* increases. Thus, *P*_D_ − *P*_C_ is minimized for the following on–off control3.2
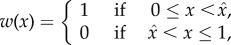
with 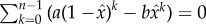. This means that rewarding is optimal when the fraction of cooperators is below 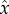; otherwise, punishing is optimal. For obvious reasons, we call this institutional sanctioning policy ‘first carrot, then stick’.

### 3.2. Effectiveness and efficiency of ‘first carrot, then stick’

**Figure 1.**
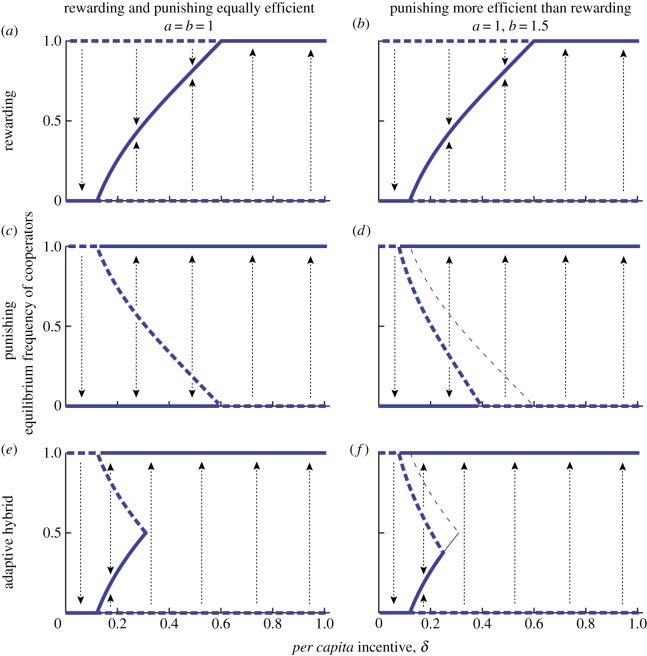
Equilibrium cooperation frequencies of public good games in well-mixed populations for three institutional sanctioning policies. Each panel shows how the locations of stable and unstable equilibrium cooperation frequencies (*continuous* and *dashed* lines, respectively) depend on the *per capita* incentive *δ*. With no or very small incentives *δ*, full defection (*x* = 0) is the only outcome, and for sufficiently large incentives *δ*, so is full cooperation (*x* = 1). This result applies to all three considered sanctioning policies: pure rewarding with *w* = 1, pure punishing with *w* = 0, and the adaptive hybrid policy with *w* given by equation (3.2). Intermediate incentives *δ* have strikingly different impacts, as follows. (*a*,*b*) *Rewarding*: When the institution increases the incentive beyond a threshold, a stable interior equilibrium enters the state space at *x* = 0, moves up to *x* = 1, and eventually exits the state space at *x* = 1. Consequently, full defection gives way to intermediate levels of cooperation, and finally to full cooperation. This outcome is independent of the initial condition. (*c*,*d*) *Punishing*: When the institution increases the incentive beyond a threshold, an unstable interior equilibrium enters the state space at *x* = 1, moves down to *x* = 0, and eventually exits the state space at *x* = 0. Consequently, full defection gives way to bistability of full defection and full cooperation, and finally to full cooperation. In (*d*), as the leverage *b* of punishing increases, full cooperation is established more readily, and the threshold value above which full cooperation is established regardless of the initial condition is smaller than in (*c*). For ease of comparison, the *thin dashed* line in (*d*) shows the unstable equilibria corresponding to (*c*). (*e*,*f*) *Adaptive hybrid*: When the institution increases the incentive beyond a threshold, full defection gives rise to bistability of intermediate cooperation and full cooperation, and finally to full cooperation. The two interior equilibria that arise, one stable and one unstable, annihilate each other for sufficiently large institutional incentives, leaving full cooperation as the global attractor. In (*f*), as the leverage *b* of punishing increases, full cooperation is established for smaller institutional incentives than in (*e*). For ease of comparison, the *thin continuous* and *dashed* lines in (*f*) show the stable and unstable equilibria of (*e*), respectively. As punishing is more effective than rewarding, the switching threshold of the adaptive hybrid policy is below *x* = 0.5. Parameters: *n* = 5, *r* = 2, *c* = 1, *a* = 1 and *b* = 1 (*a*,*c*,*e*) or 1.5 (*b*,*d*,*f*).

**Figure 2.**
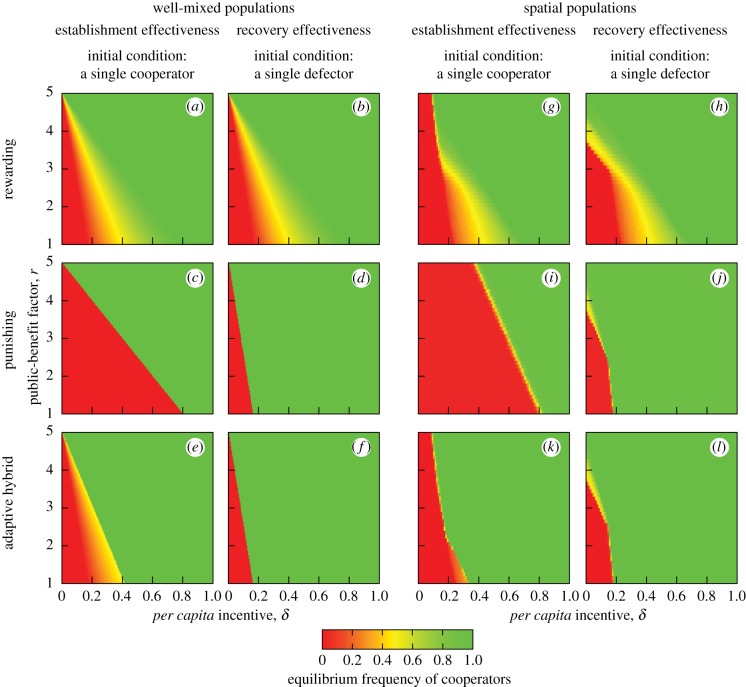
Effects of institutional sanctioning policies on public good games in well-mixed and spatial populations. The adaptive hybrid policy exhibits the broadest domain of successfully establishing full cooperation (*green*) from an initially single cooperator (first and third columns from the left), and also of recovering full cooperation against an initially single defector (second and fourth columns from the left). With no or very small *per capita* incentives *δ*, full defection (*red*) is the only outcome, and with sufficiently large incentives, so is full cooperation; this result applies to all three institutional sanctioning policies. Intermediate incentives have strikingly different impacts, as follows. (*a*,*b*,*g*,*h*) *Rewarding*: In well-mixed populations, the outcome is independent of the initial condition; (*a,b*) are identical. In spatial populations, by contrast, full cooperation and full defection are more likely to be maintained when the public-benefit factor *r* is large and the *per capita* incentive *δ* is small, as seen in the upper left corners of (*g*) and (*h*), respectively. (*c*,*d*,*i*,*j*) *Punishing*: When the institution increases *δ* beyond a threshold value (which depends on *r*), full defection abruptly changes into full cooperation. The differences between (*c*) and (*d*) or (*i*) and (*j*) indicate combinations of *r* and *δ* for which full cooperation and full defection are both stable and for which the initial conditions, therefore, affect the outcome. The differences between (*c*) and (*i*) indicate that, interestingly, spatial population structure substantially reduces the range of combinations of *r* and *δ* for which a single cooperator can invade, especially for large *r*. In (*i*)—and also in the upper parts of (*g*) and (*j*), as well as in the lower parts of (*k*) and (*l*)—the narrow (*yellow*) band between no cooperation and full cooperation results from the survival probability of the initial cooperator (and therefore does not indicate the coexistence of cooperators and defectors). (*e*,*f*,*k*,*l*) *Adaptive hybrid*: The domain of recovering full cooperation is almost equal to the case of punishing (*f,l*), while the domain of establishing full cooperation is much enlarged relative to the case of punishing, (*e*) and (*k*). In particular, as the institution increases *δ*, the equilibrium frequency of cooperators gradually rises, and when *δ* crosses a threshold value (again dependent on *r*), which is smaller than in the case of punishing, full cooperation is established abruptly (*e*,*k*). Parameters: *n* = 5, *c* = 1, *a* = *b* = 1, *s* = 10 and *N* = 100 (implying a population size of 10 000).

Figure 1 shows how the replicator dynamics are affected by *per capita* incentives *δ* under pure rewarding, pure punishing and the optimal policy in equation (3.2), with the assumption that rewarding is equally efficient (*a* = *b*) or less efficient (*a* < *b*) than punishing [32] (see also figure 2*a–f*). In particular, the effects of the optimal policy, which are illustrated in figure 1*e,f* can be understood analytically. At the boundaries *x* = 0 and *x* = 1, *P*_D_ − *P*_C_ in equation (3.1) takes the values *F* − *anδ* and *F* − *bnδ*, respectively. With the weight *w*(*x*) from equation (3.2), *P*_D_ − *P*_C_ takes its maximum value, 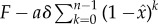 (or equivalently, 
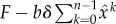
), at 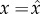; *P*_D_ − *P*_C_ is strictly increasing for 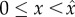 and strictly decreasing for 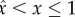. Hence, *P*_D_ − *P*_C_ = 0 can have at most two interior roots *x*. Indeed, for *δ* < min{*F*/(*an*),*F*/(*bn*)}, the replicator dynamics converge to *x* = 0. For *δ* > *F*/(*an*), a stable equilibrium enters the interior state space 0 < *x* < 1 at *x* = 0, and the full-defection equilibrium, *x* = 0, becomes unstable. For *δ* > *F*/(*bn*), an unstable equilibrium enters the interior state space 0 < *x* < 1 at *x* = 1, and the full-cooperation equilibrium, *x* = 1, becomes stable. Thus, the interior state space 0 < *x* < 1 has both stable and unstable equilibria if3.3
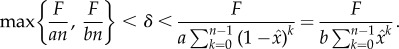
As *δ* increases within this interval, the two interior equilibria approach each other and eventually merge and vanish at the upper bound of this interval. For values of *δ* beyond this interval's upper bound, the replicator dynamics converge to *x* = 1. In the special case when rewarding and punishing are equally efficient, *a* = *b*, the stable and unstable equilibria enter the interior state space simultaneously (figure 1*e*). Conversely, in the extreme case when rewarding is much less efficient than punishing, 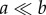, punishing alone is sufficient from the very beginning, and hybridization with relatively expensive rewarding is irrelevant.

For well-mixed populations, we have proved in §3.1 that the hybridization of positive and negative incentives according to the ‘first carrot, then stick’ sanctioning policy described by equation (3.2) minimizes the defector's advantage, which ensures that this sanctioning policy is most effective for converting a population of defectors to cooperation (figure 2*a*–*f*). By combining the differential advantages of rewarding and punishing, this sanctioning policy is far more effective than pure punishing in establishing cooperation (figure 2*c*,*e*) and far more effective than pure rewarding in recovering cooperation (figure 2*b*,*f*). Offering the ‘best of both worlds’, the ‘first carrot, then stick’ sanctioning policy for combining rewarding with punishing is therefore hereafter also called the ‘adaptive hybrid’ sanctioning policy.

Although it is natural to expect that the threshold 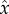 at which the adaptive hybrid policy switches from rewarding to punishing might depend on other parameters, this is not the case: the threshold remains the same independent of the *per capita* incentive *δ* and the public-benefit factor *r*. What is more, when there is no difference in leverage between positive and negative incentives (*a* = *b*), this threshold is situated at a 50% frequency of cooperators, 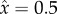. In practice, punishing is often more effective than rewarding (*a* < *b*) [32], in which case the switching point for optimal hybridization is situated at a frequency of cooperators of less than 50%, 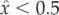 (figure 1*f*).

**Figure 3.**
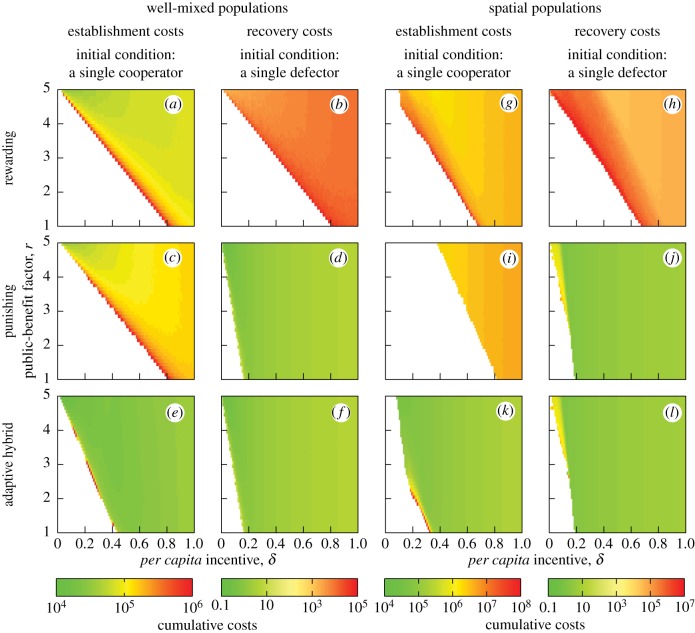
Costs for establishing and recovering full cooperation. The adaptive hybrid policy is not only the most effective (figure 2) but also the least expensive in establishing full cooperation from an initially single cooperator (first and third columns from the left) and in recovering full cooperation against an initially single defector (second and fourth columns from the left). If no or very small incentives are provided, achieving each of these goals is impossible (*white*), independent of the institutional policy. Otherwise, these policies have strikingly different impacts on the required cumulative costs. (*a*,*b*,*g*,*h*) *Rewarding*: Both in well-mixed and in spatial populations, rewarding requires recovery costs that are 1000–100 000 times more expensive than those for punishing or the adaptive hybrid policy. Furthermore, this relative cost difference increases in proportion to population size. (*c*,*d*,*i*,*j*) *Punishing*: In the case of punishing, recovery costs are much reduced relative to the case of rewarding, while establishment costs remain at a similarly high level as, or are even slightly larger than, in the case of rewarding. (*e*,*f*,*k*,*l*) *Adaptive hybrid*: The adaptive hybrid policy requires recovery costs that are similar to the case of punishing (and thus much lower than in the case of rewarding), but substantially reduces establishment costs relative to either rewarding or punishing. (For a detailed explanation of the costs at the border of the *white* regions, see electronic supplementary material, figure S1.) All parameters as in figure 2.

The adaptive hybrid policy is not only more effective, but also more efficient, for establishing and recovering cooperation than either rewarding or punishing alone (figure 3*a*–*f*). Once a state of full cooperation has been reached, punishing is cheaper as a means of recovering cooperation, as it needs to be used only occasionally. As the adaptive hybrid policy stipulates punishment once the frequency of cooperators surpasses the threshold 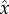, it is similar to pure punishment in this respect. The two policies differ markedly, however, in the cost of converting a population of defectors to a population of cooperators. The adaptive hybrid policy has the lowest cumulative cost of all three sanctioning policies, and hence requires both the lowest establishment cost and the lowest recovery cost for full cooperation. With respect to conversion speed, it generically takes a similar (finite) time for all three policies to establish and recover cooperation (electronic supplementary material, figure S1).

### 3.3. Extension to spatial populations

In the real world, social planning tends to be spatially distributed and is often supported by sanctioning institutions. To see whether the adaptive hybrid policy copes well with the resulting spatio-temporal complexity, we extend our framework to a spatial population in which each individual inhabits one cell of an *N* × *N* square lattice with periodic boundaries. In each generation, every individual on this lattice engages in a public good game with its four nearest neighbours (*n* = 5) and collects its total pay-off through joining all the five games within its interaction neighbourhood. Then, the strategies of all individuals are updated simultaneously. When a focal individual *i*'s strategy is updated, a neighbour of it, *j*, is drawn at random among *i*'s four nearest neighbours. Subsequently, individual *i* adopts its neighbour *j*'s strategy with probability [1 + exp(*s*(*E_i_* − *E_j_*))]^−1^, where *E_i_* denotes individual *i*'s total pay-off and *s* determines the intensity of selection [33,34]. The sanctioning institution receives feedback from the five local participants, which means that the *x* in equation (3.2) denotes the frequency of cooperators within a given neighbourhood. The positive and negative incentives determined by the adaptive hybrid policy therefore vary across the lattice as local conditions require.

**Figure 4.**
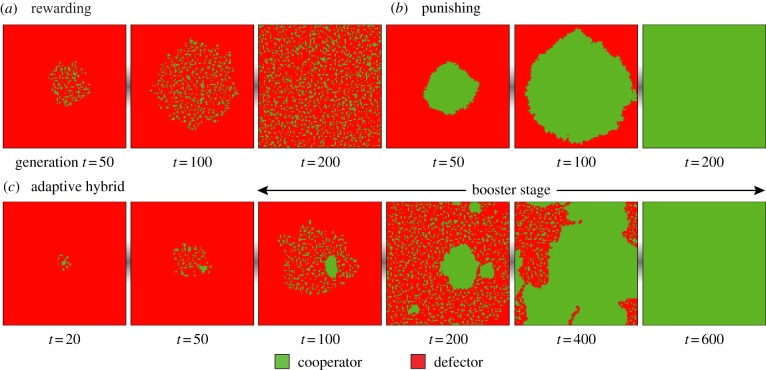
Emerging patterns of cooperation. For each incentive policy, the sequence of panels displays the spatio-temporal dynamics of cooperation, starting from a single cooperator in a population of defectors. (*a*) *Rewarding*: A fragmented cluster of cooperators interspersed with defectors expands until small cooperator clusters occur across the whole population (electronic supplementary material, movie S1). (*b*) *Punishing*: The initially single cooperator expands into a contiguous cluster of cooperators, which eventually covers the entire population (electronic supplementary material, movie S2). (*c*) *Adaptive hybrid*: The initial spread of small cooperator clusters closely resembles the case of rewarding. This development prepares the ground for local switches from rewarding to punishing, which drives the expansion of contiguous clusters of cooperators. This ‘booster stage’ enables the establishment of full cooperation for much lower incentives *δ* than in the case of punishing (electronic supplementary material, movie S3). Parameters: *r* = 2 and *δ* = 0.22 (*a*), 0.75 (*b*) or 0.22 (*c*). All other parameters as in figure 2.

Also in spatial populations, the adaptive hybrid policy turns out to be superior (figure 2*g*–*l*). Unexpectedly, it gives rise to spatial patterns of cooperation and defection that cannot easily be predicted from the patterns arising under either rewarding or punishing alone. For small and large incentives, the patterns emerging from the adaptive hybrid policy when considering a single cooperator in a population of defectors resemble the patterns observed under pure rewarding and punishing, respectively. Cooperators thrive under a policy of pure rewarding (figure 4*a*), forming fragmented islands in which they are locally interspersed with defectors, but ultimately fail to establish full cooperation for the incentive strength considered. Under a policy of pure punishing (figure 4*b*), spatio-temporal dynamics are different: an invasion that begins with a single cooperator in a population of defectors always results in a contiguous cluster of cooperators that grows and eventually displaces all defectors. The adaptive hybrid policy, by contrast, for intermediate incentive strengths exhibits an intriguing transition between these two distinct patterns: fragmented islands of cooperators, initially supported by rewarding, create circumstances under which punishing can act as a ‘booster stage’ that capitalizes on and amplifies the pro-social effects of rewarding, promoting the rapid growth of contiguous clusters of cooperators that eventually displace all defectors (figure 4*c*).

All three policies are capable of recovering cooperation in much the same way as for well-mixed populations. The only qualitative difference is that the successful spread of defectors originating from an initially single defector can occasionally split contiguous clusters of cooperators. This phenomenon, however, which has previously been described for the spatial extension of the well-studied Prisoner's Dilemma [35], occurs in our model only for vanishing or very small incentives.

### 3.4. Robustness

In the electronic supplementary material, we demonstrate the robustness of our results with respect to the following model variants. (i) First, we establish that in spatial populations the adaptive hybrid policy with either local or global feedback establishes and recovers full cooperation at lower cost and under a wider range of conditions than an alternative hybridization of positive and negative incentives in which the weight *w* is proportional to the frequency of cooperators, *w*(*x*) = *x* (electronic supplementary material, figure S2). We also show that information about the local frequency of cooperation allows a sanctioning institution that implements the adaptive hybrid policy to establish full cooperation more readily than information about the global (i.e. population-wide) frequency of cooperation [20]. This result is in line with expectations, as tailoring a strategy to local conditions should generally achieve better results than a strategy that depends on conditions averaged across large spatial scales.

We also explore (ii) a variant of the public good game in which a cooperator does not benefit from its own contribution [4,19] (electronic supplementary material, figure S3) and (iii) a variant of the incentive scheme in which we relax the assumption that the received incentive is inversely proportional to the number of cooperators or defectors in an interacting group [4,19] (electronic supplementary material, figure S4). Furthermore, we test variants of our spatial model with (iv) interactions encompassing the eight nearest neighbours [33–35] (chess-king move, *n* = 9, electronic supplementary material, figure S5), (v) smaller population size (electronic supplementary material, figure S6), (vi) asynchronous updating [33,34] (electronic supplementary material, figure S7), (vii) a proportional imitation rule [33,34] (electronic supplementary material, figure S8), (viii) errors in perception and implementation (for individuals [36] or institutions [37], electronic supplementary material, figures S9–S13) and (ix) varied switching thresholds (electronic supplementary material, figure S14). We find that none of the variants (ii)–(viii) qualitatively affect our results regarding the effectiveness and efficiency of the three considered sanctioning policies (electronic supplementary material, figures S3–S13). Exploring (ix) reveals that, for equal leverages of positive and negative incentives, the optimal switching threshold for spatial populations approximately equals 50%, just as in well-mixed populations (electronic supplementary material, figures S14).

As a final model variant, we assume that (x) individuals equally share the cost of funding the incentive budget (e.g. through an entrance fee or poll tax) [4,19]. For this model variant, we find the resulting dynamics to be entirely unaffected.

## 4. Discussion

Here we have demonstrated how an institutional sanctioning policy of ‘first carrot, then stick’ can be surprisingly successful in promoting cooperation. This policy establishes and recovers cooperation at a lower cost and under a wider range of conditions than either rewards or penalties alone can do. Our findings are based on the public good game, a standard framework for cooperation in groups. They apply to both well-mixed and spatial populations and remain robust under a broad spectrum of model variations and parameter combinations.

To promote cooperation, rewards and penalties are frequently used in concert. Considering how often they are used together—at all levels of organization, from parents to teachers to leaders of organizations—it is surprising that no prior study to date has investigated the optimal use of a combination of rewards and penalties in an institutional setting. Here we have demonstrated that the optimal institutional sanctioning policy is not given by a gradual change in the relative allocation towards rewards and penalties, but by a sudden switch from positive to negative incentives once cooperation is sufficiently widespread. For example, teachers who wish to establish order in an unruly group of pupils may thus be well advised first to reward those pupils who behave well and, once good behaviour has become the norm, shift to reprimanding those pupils who do not. Similarly, leaders faced with the difficult task of implementing an unpopular directive may initially reward those who voluntarily comply with it and, once the directive has been largely phased in, make it mandatory, enforced by a penalty for non-compliance. As these two examples indicate, the ‘first carrot, then stick’ sanctioning policy can easily be adapted to many real-life situations, making it a widely applicable method for promoting cooperation.

Interestingly, when the ‘first carrot, then stick’ sanctioning policy, here also called the adaptive hybrid policy, is used to promote cooperation in spatial populations, it gives rise to complex spatial patterns of cooperators and defectors that differ qualitatively from the simpler patterns that arise when rewards or penalties are used in isolation. This is because punishing acts as a booster stage that reinforces the pro-social effects of rewarding, thus allowing cooperation to be rapidly established in those parts of a population where cooperation has surpassed the critical threshold. Although our analytical methods do not extend to spatial populations, extensive numerical investigations confirm that a sudden switch from rewarding to punishing, not a gradual change in the relative allocation to these incentives, also is optimal for promoting and recovering cooperation in spatial populations.

Our theoretical results can be compared with the handful of experimental studies that have explored the combined use of positive and negative incentives in peer-to-peer sanctioning [38–41] or in sanctioning by an assigned team leader [42]. Although these studies differ significantly in their experimental design, they share two common characteristics. First, punishment is typically more effective than rewarding at promoting high contributions to the public good. Second, players initially have a propensity for rewarding cooperation, which is soon superseded by a propensity for punishing defectors [38–40]. While the latter trend might superficially be interpreted as corroborative evidence for the effectiveness of the institutional sanctioning policy developed here, the rationale for shifting from positive to negative incentives is strikingly different. In the experimental studies, this shift typically coincides with declining average contributions and can thus be interpreted as a response to the emergence of defectors [42]. In particular, a study on team leadership concludes that ‘leaders who experience frequent complete free-riding and high variance in contributions in their teams are more likely to switch from positive to negative incentives’ [42], while other studies find that punishing is more effective than rewarding at staving off complete free-riding [38–40]. By contrast, we have demonstrated the advantage of shifting from positive to negative incentives as contributions increase, and we predict that rewarding is more effective than punishing in staving off complete free-riding [43].

We have determined the optimal sanctioning policy for a social institution charged with overseeing rational agents. Two complementary studies on peer-to-peer sanctioning that account, respectively, for reputation effects and for the potential of group selection have similarly highlighted the role of positive incentives in promoting incipient cooperation among defectors [36,44]. These theoretical predictions, derived under the assumption of rational behaviour, clearly question the wisdom of the human behaviour observed in the aforementioned experimental studies. Understanding whether punishment in the face of rampant defection is a human fallacy or a rational choice under circumstances other than the ones analysed here is a key challenge for future research.

## Supplementary Material

Electronic supplementary material, text S1 and S2 and figures S1-S14
