# Supplementary material for: First carrot, then stick: how the adaptive hybridization of incentives promotes cooperation
Source: J R Soc Interface. 2015 Jan 6;12(102):20140935. doi: 10.1098/rsif.2014.0935 (PMC4277083; doi:10.1098/rsif.2014.0935)
Supplement: Electronic supplementary material, text S1 and S2 and figures S1-S14 [file rsif20140935supp1.pdf]

Electronic supplementary material for

First carrot, then stick: How the adaptive hybridization of incentives promotes cooperation

Xiaojie Chen, Tatsuya Sasaki, Åke Brännström, and Ulf Dieckmann

Supplementary text and figures

In §S1, we present the individual-based model for our numerical investigations. In §S2, we investigate the conversion time to full cooperation, i.e. how long it takes to establish or recover full cooperation (figure S1). To examine the robustness of our main results, we finally, in §S3, consider a range of variants of our main model (figures S2–S14).

S1. Individual-based model

In our individual-based model, the population is updated synchronously. In each generation, every individual collects its total pay-off through joining a fixed number of games (in well-mixed populations, a single game with $n-1$ randomly selected co-players, or in spatial populations, all possible games within its interaction neighbourhood). Then, the strategies of all individuals are updated simultaneously. When individual i's strategy is updated, a neighbour j is drawn at random (in well-mixed populations, from the entire population, or in spatial populations, among all of i's $n-1$ neighbours). Subsequently, individual i adopts its neighbour j's strategy with probability $[1+\exp(s(E_i-E_j))]^{-1}$, where E_i denotes individual i's total pay-off and s determines the intensity of selection [33,34]. Cumulative costs in (finitely large) well-mixed populations (figure 3) are calculated by means of individual-based model runs.

For spatial evolutionary games, it is well known that contiguous clusters of cooperators often fare well in a population dominated by defectors, in which an isolated cooperator would achieve a pay-off well below the population average [35]. Our numerical investigations respect the latter, most stringent, initial condition. To study the conditions under which full cooperation can be stabilized, we further consider the other extreme: a single defector in a population of cooperators. For infinite and well-mixed populations, 'a single cooperator/defector' (e.g. in figure 2) means an infinitesimally small fraction of cooperators/defectors. For finite spatial populations, the equilibrium frequency of cooperators is determined as the fraction of cooperators in the whole population in the stationary state reached after sufficiently many generations. Each data point depicted in figures 2 and 3 represents the mean of 100 independent model runs.

S2. Conversion to full cooperation: establishment and recovery

To better understand the factors that cause differences in cumulative costs between institutional sanctioning policies, we consider the conversion time to full cooperation. Specifically, by using the individual-based model (§S1) we investigate the time required (i) to establish full cooperation from an initially single cooperator, and (ii) to recover full cooperation after a single defector has entered the population. Although it seems natural that these times would change significantly with parameters settings, it turns out that they are largely independent of the public-benefit factor and the size of the institutional incentive, except when these are reduced to values just above the threshold at which full cooperation can be established and recovered. In these cases, it can take an exceptionally long time to establish and recover cooperation, as seen in figure S1.

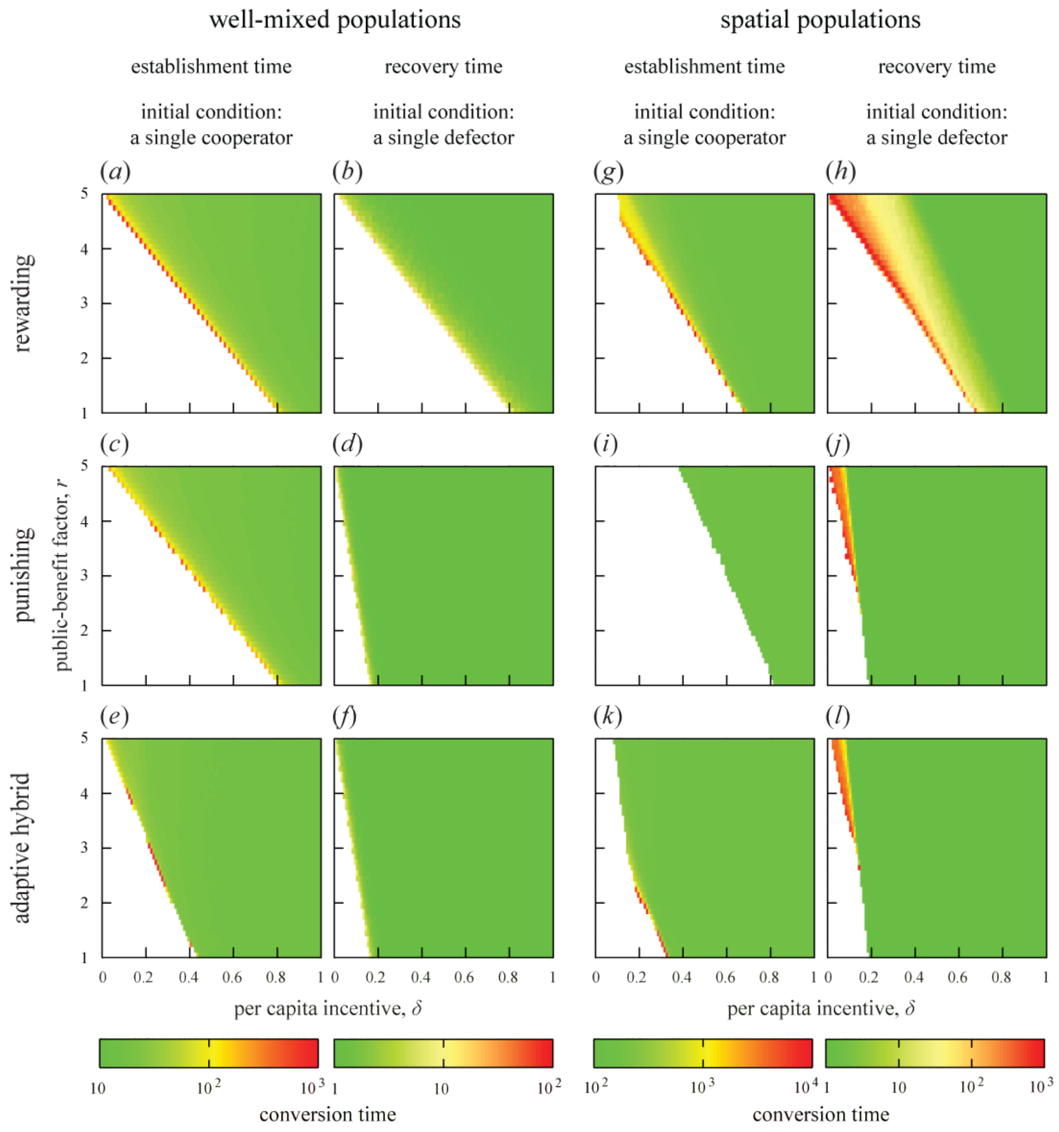

Figure S1. Establishment and recovery time. The establishment time describes how long it takes to achieve full cooperation from an initially single cooperator in a population of defectors. Similarly, the recovery time describes how long it takes to resume full cooperation when a single defector is introduced into a population of cooperators. The times required to establish and recover cooperation are on the same order of magnitude for all three institutional sanctioning policies, except when the public-benefit factor and the per capita incentive are reduced to values just above the threshold at which full cooperation can be established and recovered. This is particularly visible for the recovery time in spatial populations (*h*). Note that rewarding is the most expensive policy for recovering cooperation. Parameters that are not varied are the same as in our main model (figure 2).

S3. Alternative model variants

For spatial populations—and, in cases (ii) and (iii) below, also for well-mixed populations—we investigate a range of model variants in which we change (i) the weight function and the feedback rule, (ii) the specification of the public good game, (iii) the incentive scheme, (iv) the interaction neighbourhood, (v) the population size, (vi) the population update scheme, (vii) the individual update rule, (viii) individual and institutional errors of perception and implementation, and (ix) the switching threshold for the on-off control. In particular, in (viii) we explicitly address a challenge highlighted in a recent review by Simon Gächter: 'real-life institutions do not work perfectly; for example, punishment or reward may not be correctly implemented. It remains unclear how these imperfectly applied incentives might affect cooperation' [37].

(i) Weight function and feedback rule. We compare four variants of the spatial model in which the feedback is either local, from the interaction neighbourhood, or global, from the whole population (as in the case of well-mixed populations), and in which the weight function w is either the on-off control or a linear control under which w is given by the frequency x of cooperators. We find that, among those four variants, the on-off control with local feedback is most widely successful, as well as least expensive, at promoting full cooperation (figure S2).

(ii) Public good game. We consider a variant of the public good game, called 'others-only' [4,19]. Here, a player's contribution is shared equally among the other $n-1$ co-players in an n-player group, so that contributors have no direct gain from their own investments. In this variant, the public good game constitutes a social dilemma, independent of the public-benefit factor r, because a player can always improve her or his pay-off by withholding a contribution. In a group of n_{C} cooperators and n_{D} defectors (with $n_{\mathrm{C}}+n_{\mathrm{D}}=n$), the pay-offs for a cooperator and a defector are $rc(n_{\mathrm{C}}-1)/(n-1)-c+awn\delta/n_{\mathrm{C}}$ and $rcn_{\mathrm{C}}/(n-1)-b(1-w)n\delta/n_{\mathrm{D}}$, respectively. Because there is no change in the last terms of these expressions, describing the effects of incentives, equations (2.4), (2.5), (3.1), and (3.2) remain valid with the only change being that F now equals c. In contrast to our main model, the others-only variant has the property that F is constant with respect to r, and thus, so are also the thresholds δ_{-} and δ_{+}. Hence, the replicator dynamics in equation (2.2) are independent of r, and the equilibrium frequencies of cooperators change only with respect to δ (figure S3−1*a*–*f*). Our main result, that the adaptive hybrid policy of using rewards and penalties is most efficient at establishing cooperation, holds also for the others-only variant in well-mixed populations, as well as in spatial populations (figure S3−1*g*–*l*). In the latter case, in striking contrast to our main model, larger values of r make cooperation less likely to evolve through punishing (figure S3−1*i*).

(iii) Incentive scheme. We investigate a variant of the institutional sanctioning policy in which the magnitude of incentives is not inversely proportional to the number of recipients [4]. This is intended to mimic real-life situations in which the expected incentive is constant or near-constant as long as the number of recipients is not unmanageably large. Following earlier work on positive and negative incentives [4], we generalize the expected penalty as $b(1-w)n\delta/[(1-h)+h(n-n_\mathrm{C})]$, and the expected reward as $awn\delta/[(1-h)+hn_\mathrm{C}]$. In these expressions, the parameter h controls how the magnitude of incentives changes with the number of recipients, $0 \le h \le 1$. When $h=0$, incentives are independent of the number of recipients, whereas for increasing values of h, incentives become less uniform. Our main model corresponds to the case $h=1$. Our result is qualitatively unchanged for $h=$ 0.8, 0.6, 0.4, or 0.2 in the establishment case, and for $h=$ 0.8 or 0.6 in the recovery case. For much smaller values of h, the advantage of the adaptive hybrid policy is lessened, and pure rewarding and pure punishing are similarly effective at achieving a high level of cooperation for a broad range of parameters (figures S4–1 and S4–3). The adaptive hybrid policy, however, still remains least expensive, except on the verge of full cooperation (figures S4–2 and S4–4).

(iv) Interaction neighbourhood. We investigate a variant for spatial populations in which interactions occur in the Moore neighbourhood [33,34], so a focal individual interacts with individuals in the eight nearest cells (which might be reached by one move of a chess king). Individual-based model runs confirm that our main result is qualitatively unaffected by this enlarged neighbourhood (figure S5).

(v) Population size. We reduce the size of the square lattice on which individual-based model runs take place from 100×100 to 10×10, and find that our main result is qualitatively robust under such downsizing (figure S6).

(vi) Population update scheme. As a further variant for spatial populations, we consider asynchronous updating [33,34], in which in each time step one individual is chosen at random from the population, and immediately updated. Our main result, derived for synchronous updating (§S1), is robust under such asynchronous updating (figure S7).

(vii) Individual update rule. We also consider a variant of the individual update rule, in which a focal individual i with total pay-off E_i adopts the strategy of an individual j with total pay-off E_j, to whom the focal individual is being compared, with a probability proportional to their pay-off difference [33,34], provided $E_j - E_i > 0$. Specifically, the imitation probability is given by

$$\theta_{i\to j} = \begin{cases} (E_j - E_i)/\Delta & \text{if } E_j - E_i > 0, \\ 0 & \text{otherwise,} \end{cases} \tag{S1}$$

in which the scaling factor Δ ensures that $0 \le \theta_{i\to j} \le 1$. Our main result remains robust under this variation (figure S8), and changing the value of Δ does not affect the equilibrium frequency of cooperators (instead, it only scales the resulting cumulative cost).

(viii) Individual and institutional errors. We investigate the effects on our main result of five different types of possible errors in perception and implementation for individuals [36] and institutions [37]. None of the errors considered qualitatively affects our findings:

- Strategy-implementation error (figure S9). When an individual participates in the public goods game, it uses the strategy opposite to its own with probability u_1.
- Strategy-observation error (figure S10). To gauge incentives, the institution must observe each individual's strategy. We assume that, with probability u_2, the institution incorrectly observes an individual's strategy.
- Incentive-distribution error (figure S11). After the public good game, the institution applies incentives to the target individuals in a group. We assume that each of them correctly receives their respective incentive independently with probability $1 - u_3$; otherwise, the incentive is randomly given to another individual in the group. Consequently, a cooperator (defector) may be punished (rewarded), and multiple instances of rewarding and/or punishing may occur for a single individual.
- Payoff-observation error (figure S12). Each individual reviews its strategy by using a stochastic update rule following the so-called Fermi function, which depends on the pay-off difference between the focal individual and a randomly selected neighbour (§S1). In this function, we may reduce the strength s of selection to represent an increasing degree of erroneous perception of a neighbour's total pay-off.
- Strategy-imitation error (figure S13). Depending on the individual update rule, each individual makes a decision on whether or not to imitate a neighbour's strategy. If an individual decides to do so, we assume that it mistakenly imitates the wrong strategy with probability u_5. In contrast to the four aforementioned sources of errors, we find that such strategy-imitation errors facilitate the establishment of cooperation from an initially single cooperator in a population of defectors, which becomes successful for larger values of the public-benefit factor r, in particular, in the case of punishing (figure S13–1c).

(ix) Switching threshold. Finally, we explore which switching threshold for the on-off control most efficiently promotes cooperation in the spatial model. We address this question for interactions with the four nearest neighbours (figures S14–1 and S14–2), as well as for interactions with the eight nearest neighbours (figures S14–3 and S14–4). In both cases, the institution switches from rewarding to punishing when the number of cooperators in a group exceeds a given threshold $\hat{n}$. We find that for $\hat{n} = 2$ in the former case ($n = 5$) and for $\hat{n}$ around 4 in the latter case ($n = 9$), the spatial on-off control most effectively establishes full cooperation from an initially single cooperator. This result closely matches predictions of the analytical theory, $\hat{n} = 2.5$ and $\hat{n} = 4.5$, respectively.

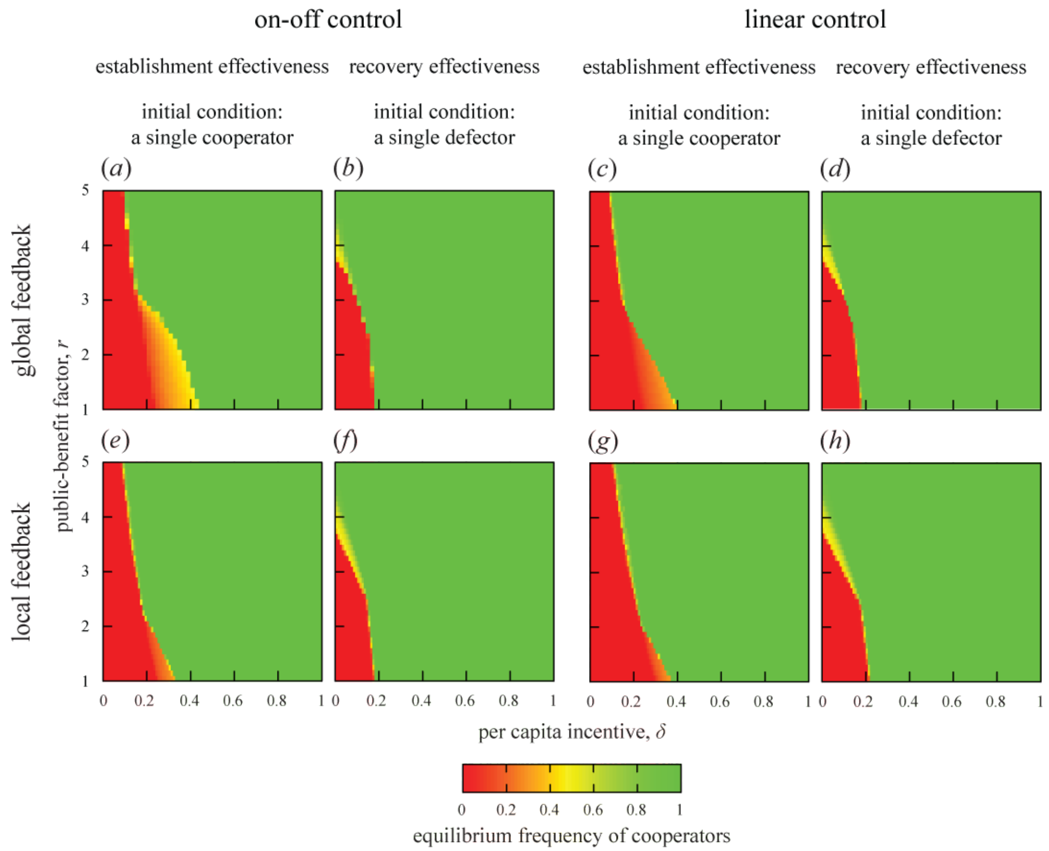

Figure S2-1. Effects of institutional sanctioning policies on spatial public good games under different feedbacks and hybridizations of incentives. The on-off control based on local feedback (*e*) is most effective for establishing and recovering cooperation. This control and feedback are used by the adaptive hybrid policy. Parameters that are not varied are the same as in figure 2.

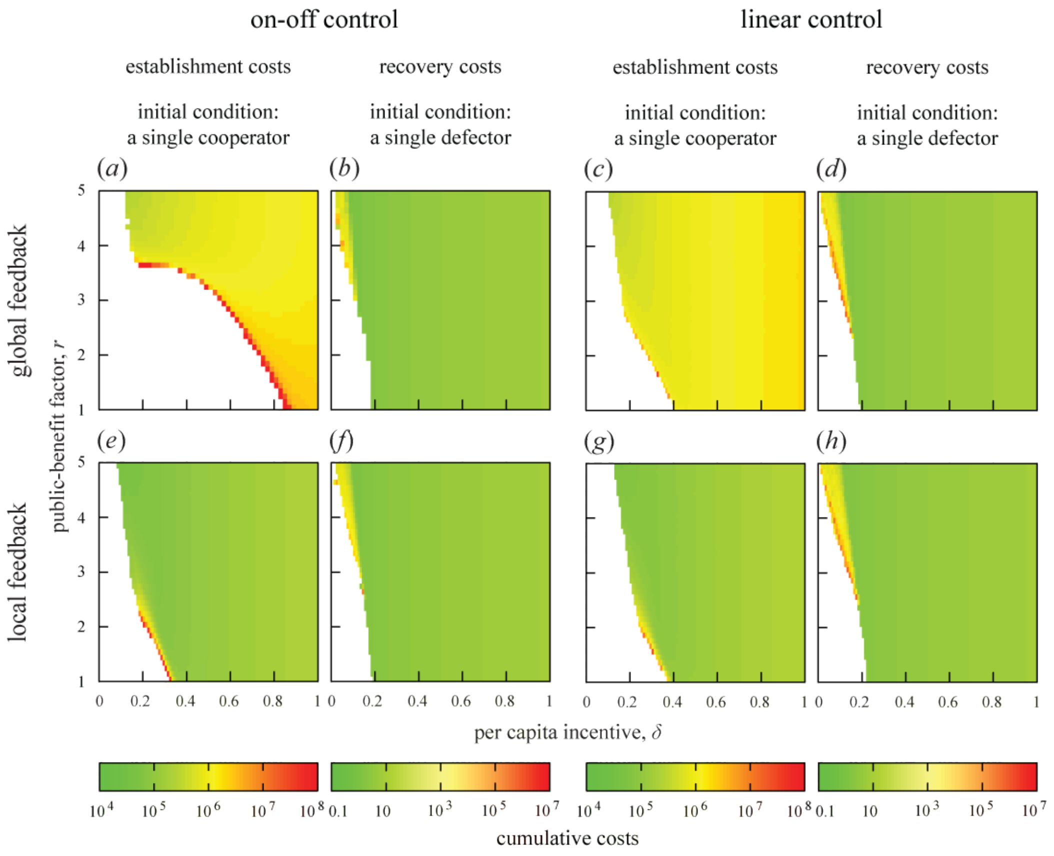

Figure S2-2. Costs for establishing and recovering full cooperation in spatial public good games under different feedbacks and hybridizations of incentives. The on-off control based on local feedback (*e*) is the least expensive option for establishing and recovering cooperation. This control and feedback are used by the adaptive hybrid policy. Parameters that are not varied are the same as in figure 2.

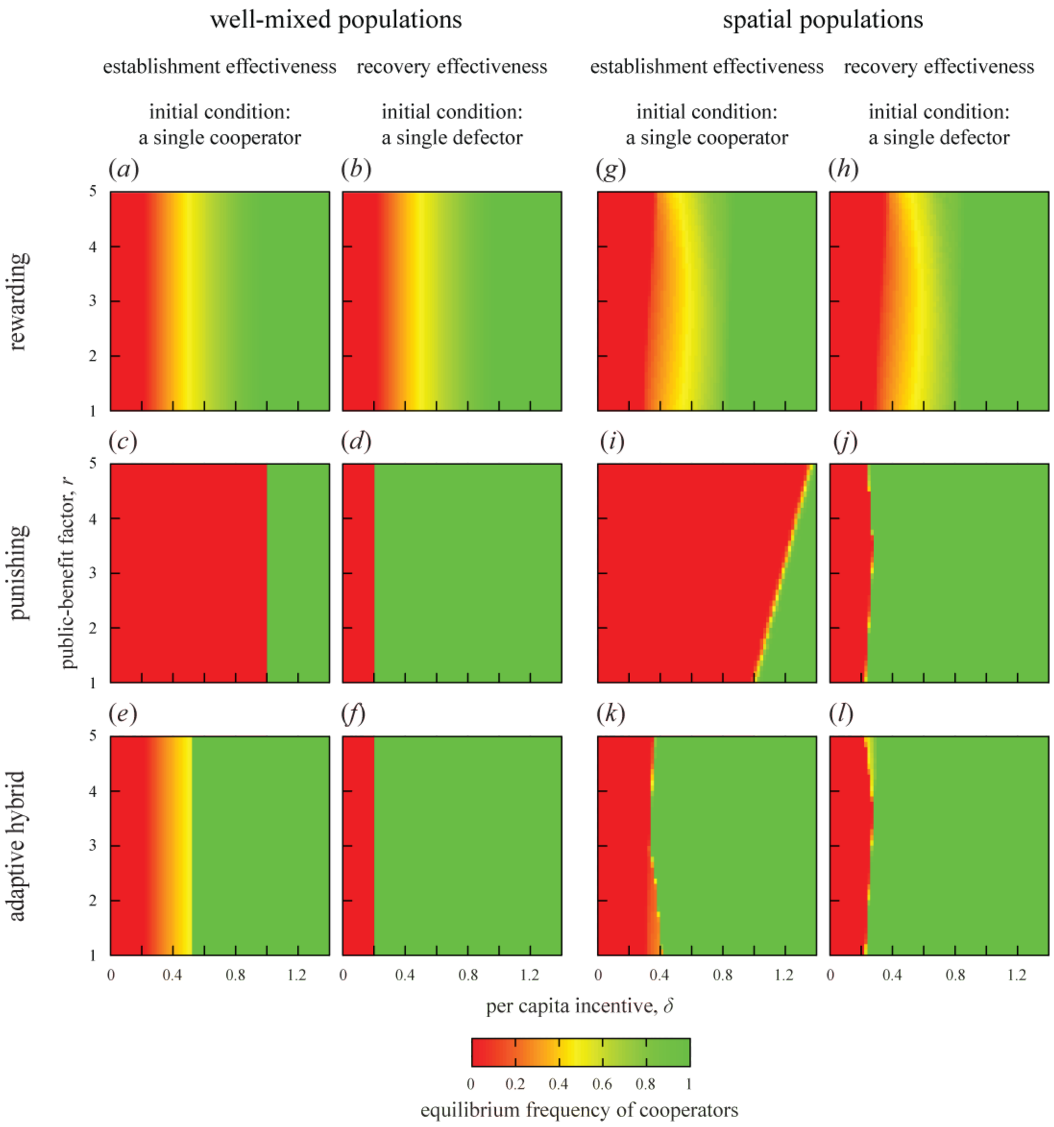

Figure S3-1. Effects of institutional sanctioning policies on 'others-only' public good games. The adaptive hybrid policy has the broadest domain of success also when individuals do not receive any direct benefits from their own investments. Note that in (*i*) the line that separates the parameter regions for full defection (*red*) and full cooperation (*green*) has positive slope, whereas, in our main model this boundary has negative slope (figure 2*i*). Parameters that are not varied are the same as in figure 2.

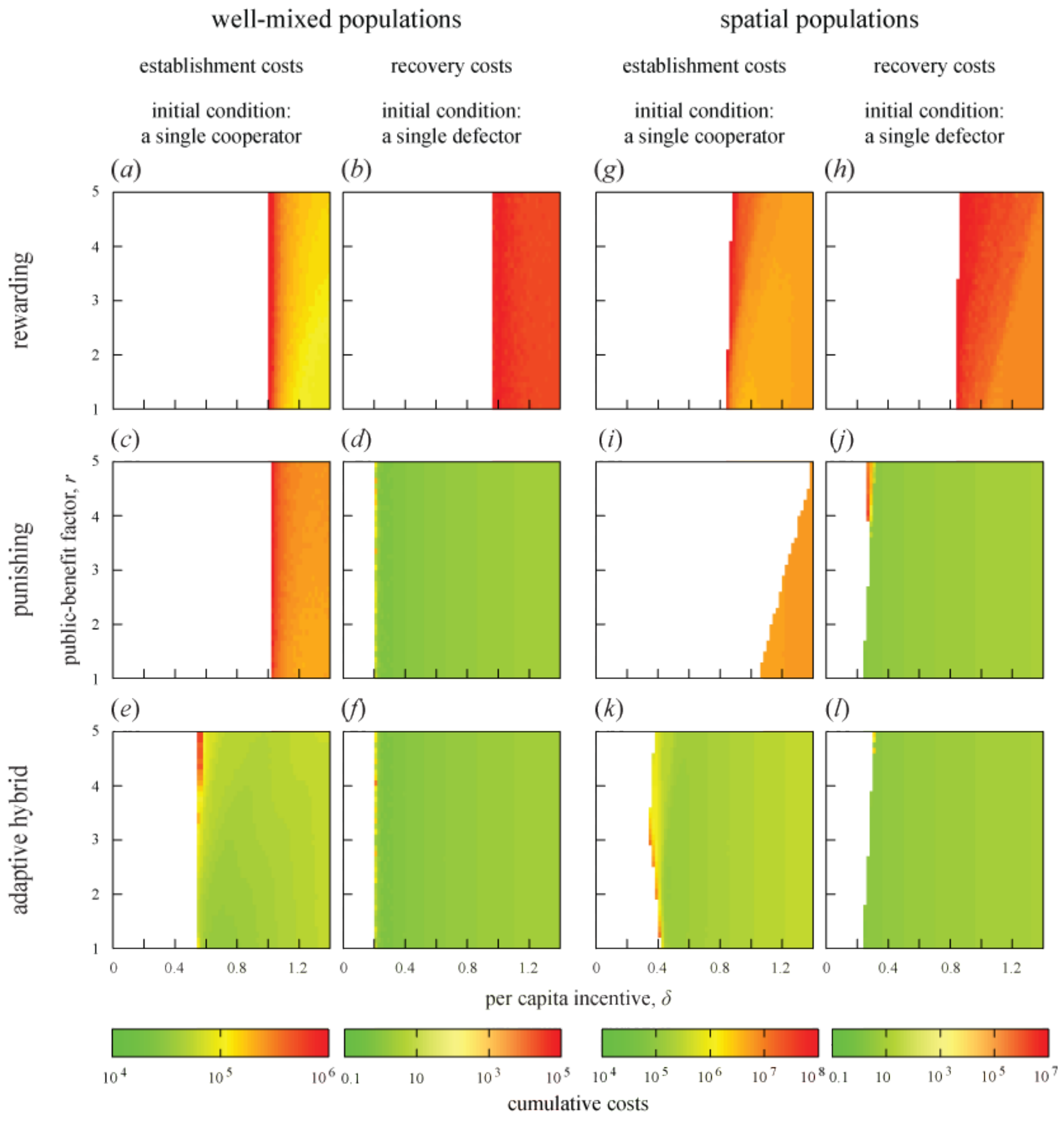

Figure S3-2. Costs for establishing and recovering full cooperation in 'others-only' public good games. The adaptive hybrid policy is the least expensive also when individuals do not receive any direct benefits from their own investments. Parameters that are not varied are as in figure 2.

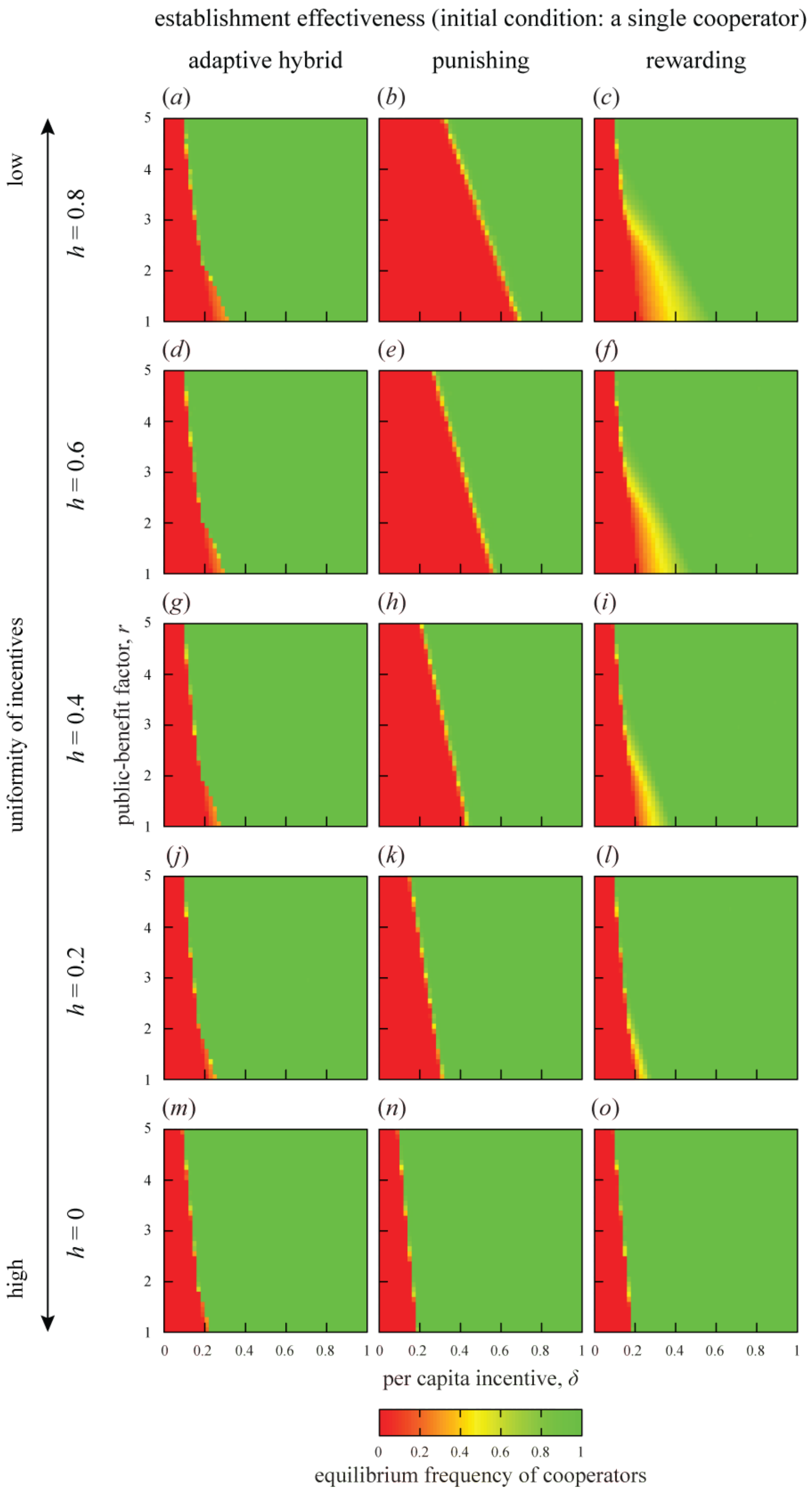

Figure S4-1. Effects of institutional sanctioning policies on spatial public good games when the magnitudes of incentives are uniform. For decreasing values of h, the magnitudes of incentives become more uniform. The adaptive hybrid policy has the broadest domain of success in establishing cooperation also when the magnitudes of incentives are not inversely proportional to the number of recipients in a focal individual's interaction group. Parameters that are not varied are the same as in figure 2.

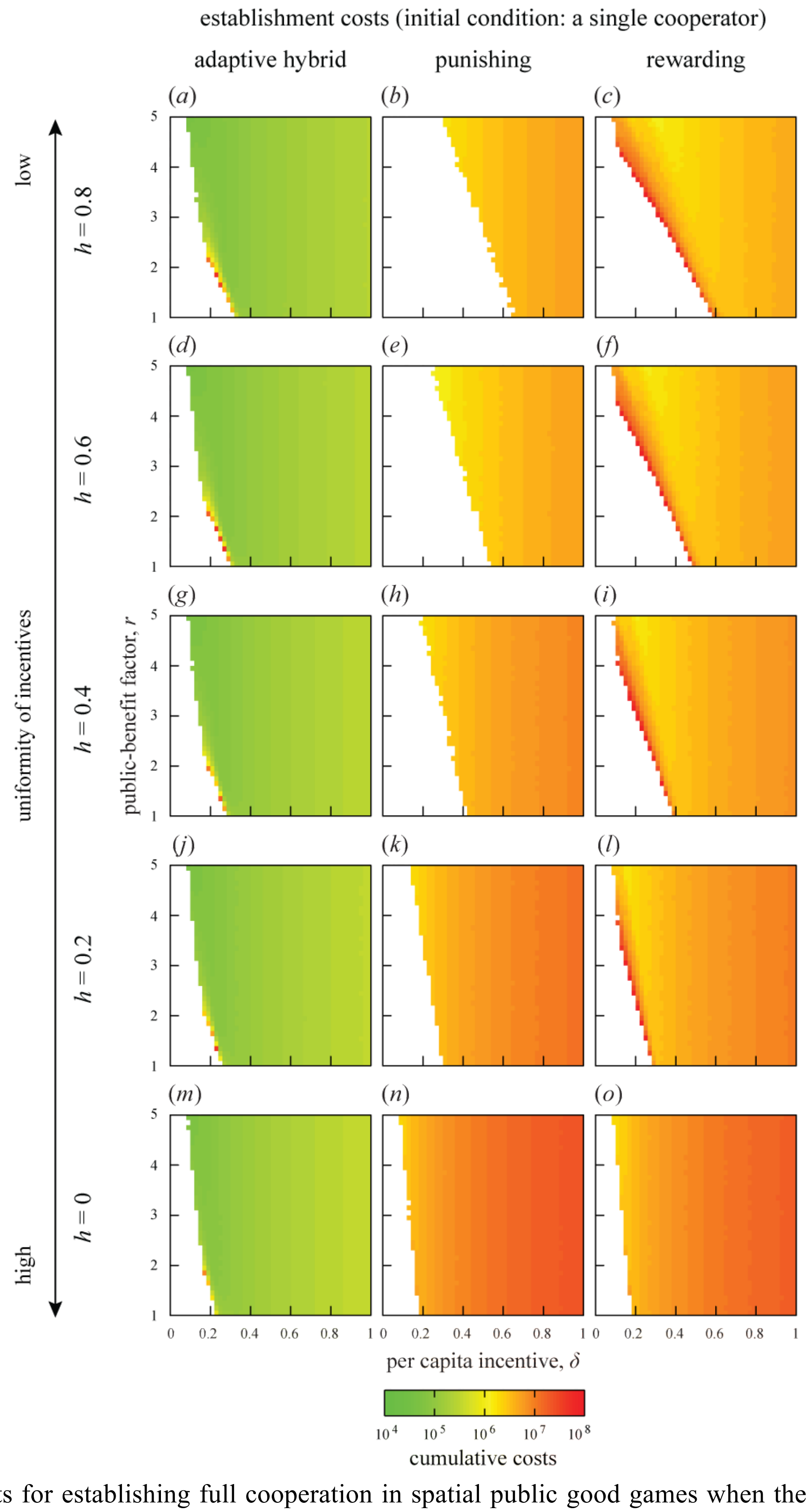

Figure S4-2. Costs for establishing full cooperation in spatial public good games when the magnitudes of incentives are uniform. For decreasing values of h, the magnitudes of incentives become more uniform. The adaptive hybrid policy remains least expensive also when the magnitudes of incentives are not inversely proportional to the number of recipients in a focal individual's interaction group. The only minor exceptions occur at the verge of full cooperation. Parameters that are not varied are the same as in figure 2.

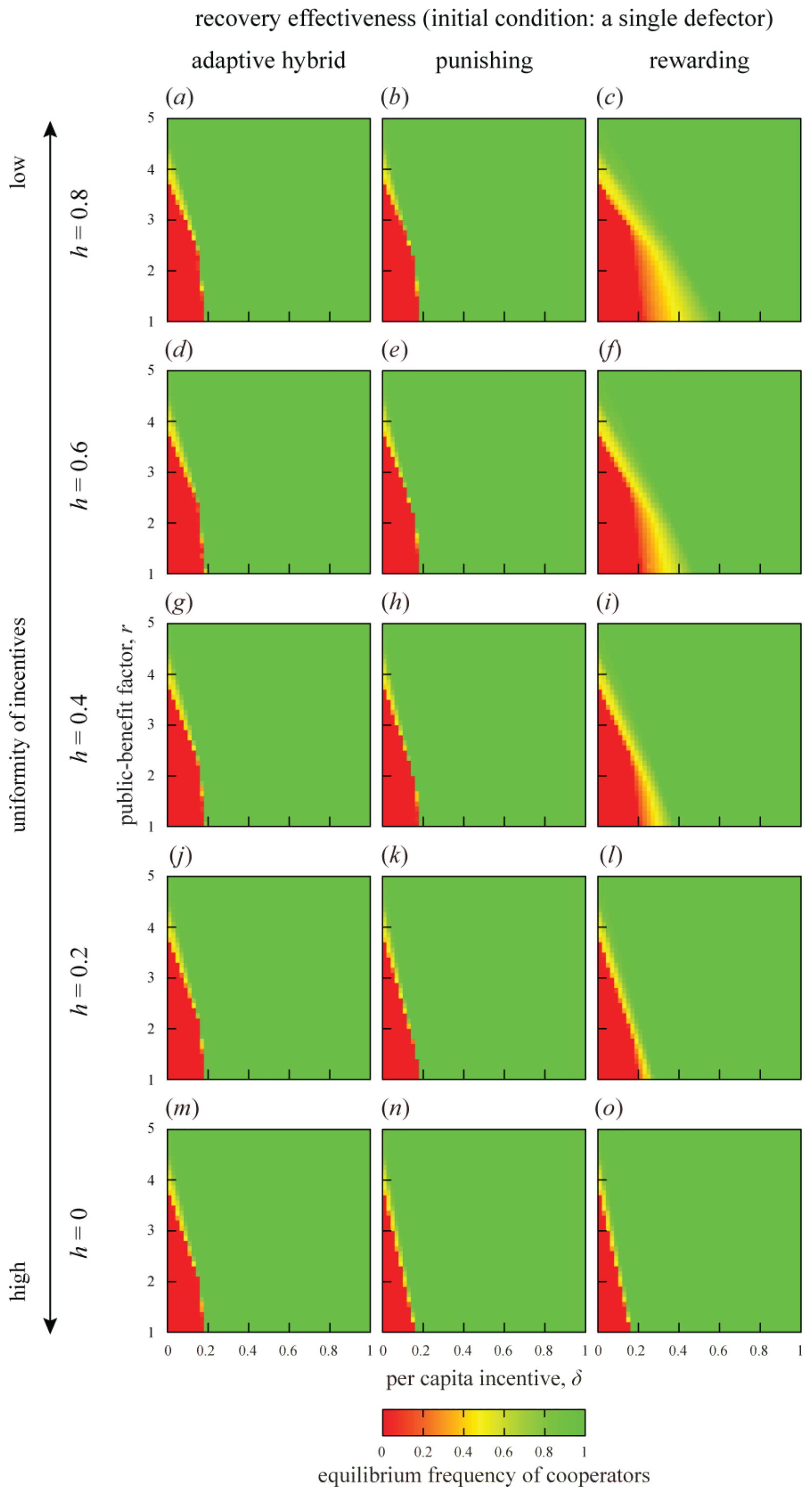

Figure S4-3. Effects of institutional sanctioning policies on spatial public good games when the magnitudes of incentives are uniform. For decreasing values of h, the magnitudes of incentives become more uniform. The adaptive hybrid policy has the broadest domain of success in establishing cooperation also when the magnitudes of incentives are not inversely proportional to the number of recipients in a focal individual's interaction group. Parameters that are not varied are the same as in figure 2.

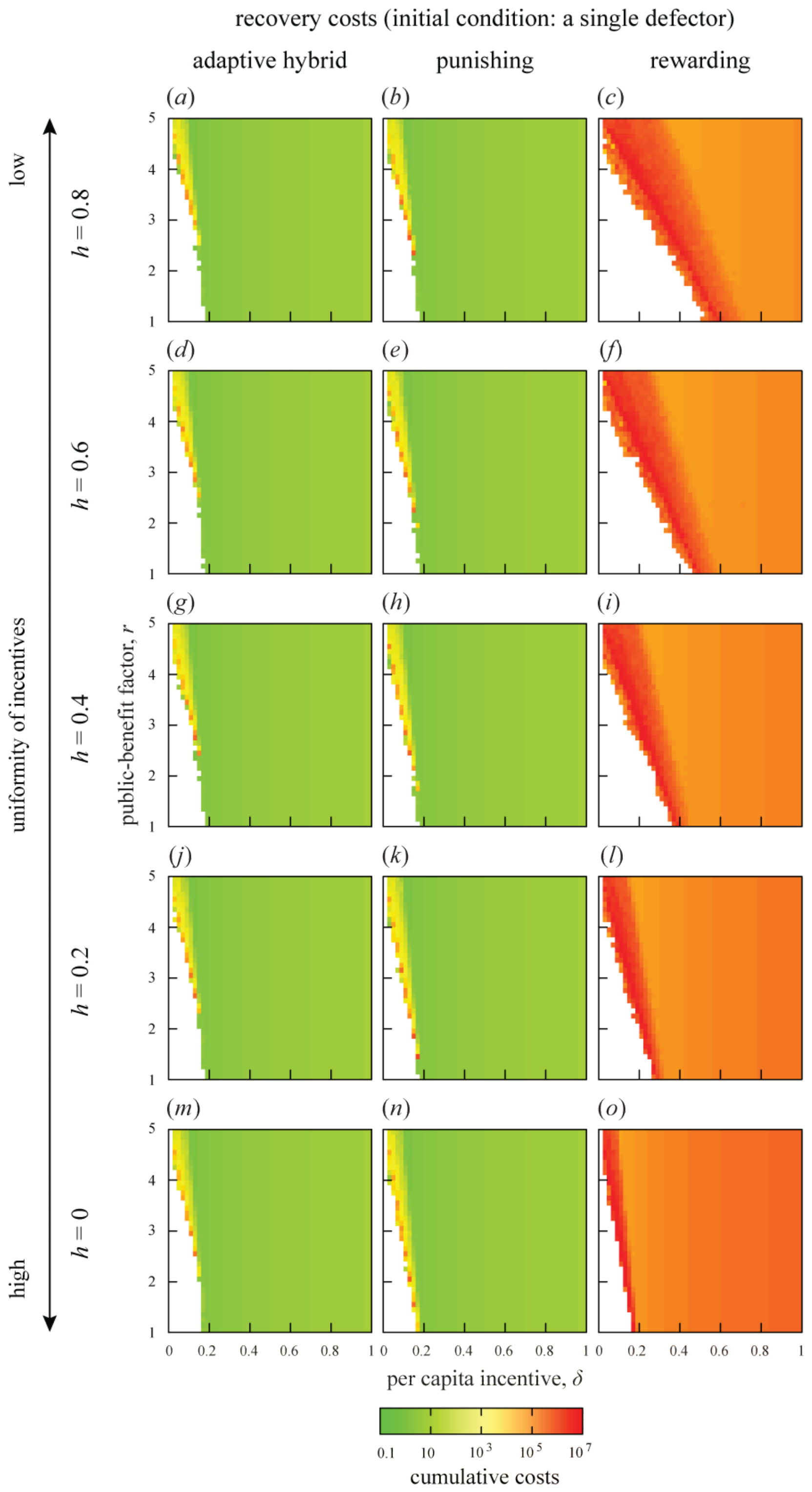

Figure S4-4. Costs for recovering full cooperation in spatial public good games when incentives are uniform in size. For decreasing values of h, the magnitudes of incentives become more uniform. The adaptive hybrid policy remains least expensive also when the magnitudes of incentives are not inversely proportional to the number of recipients in a focal individual's interaction group. The only minor exceptions occur at the verge of full cooperation. Parameters that are not varied are the same as in figure 2.

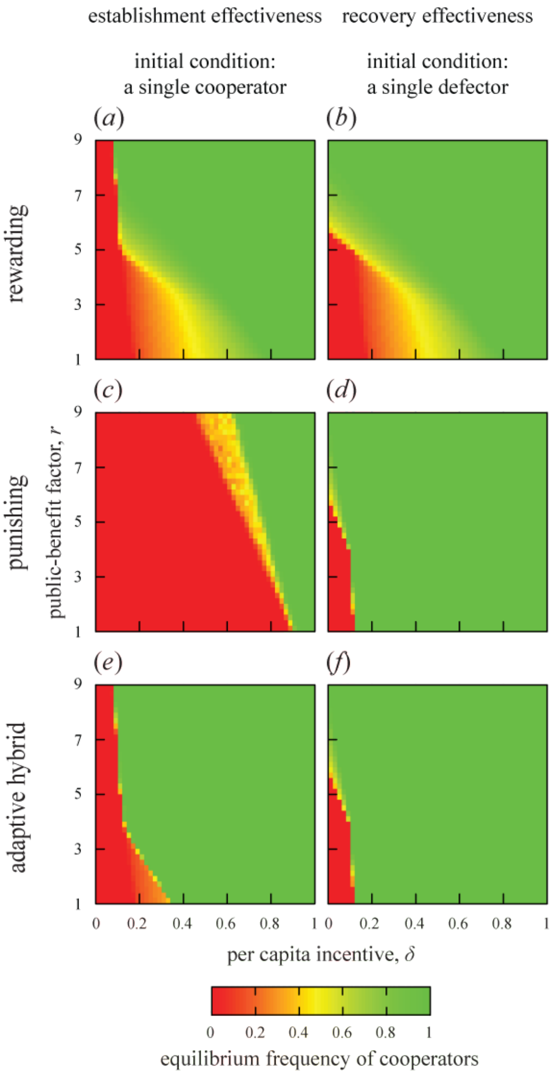

Figure S5-1. Effects of institutional sanctioning policies on spatial public good games with enlarged interaction neighbourhood. The adaptive hybrid policy has the broadest domain of success also when individuals interact with their eight nearest neighbours ($n = 9$). Parameters that are not varied are the same as in figure 2.

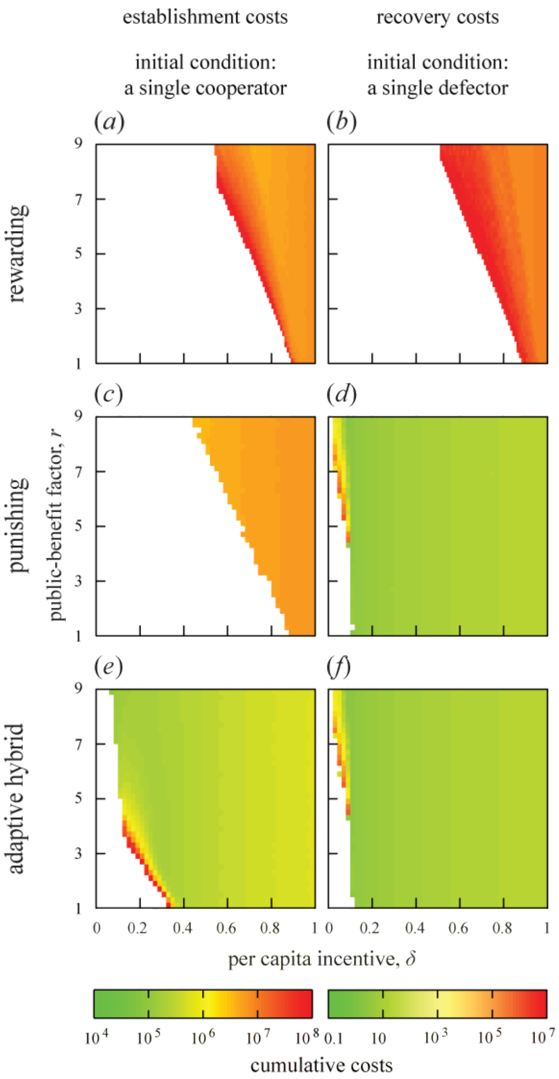

Figure S5-2. Costs for establishing and recovering full cooperation in spatial public good games with enlarged interaction neighbourhood. The adaptive hybrid policy is the least expensive also when individuals interact with their eight nearest neighbours ($n = 9$). Parameters that are not varied are the same as in figure 2.

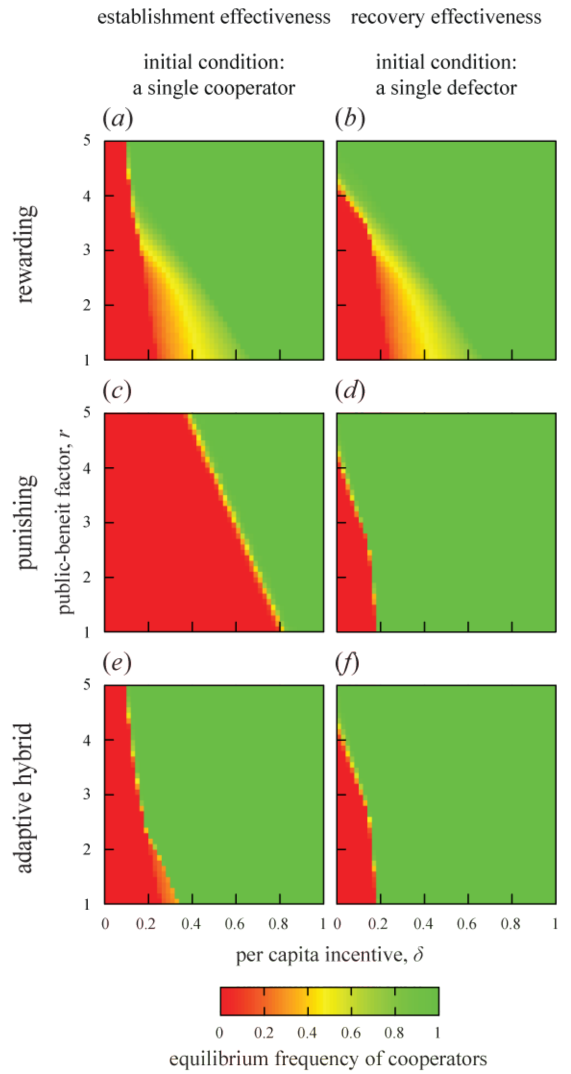

Figure S6-1. Effects of institutional sanctioning policies on spatial public good games with smaller population size. The adaptive hybrid policy has the broadest domain of success also for populations on a 10×10 periodic square lattice. Parameters that are not varied are the same as in figure 2.

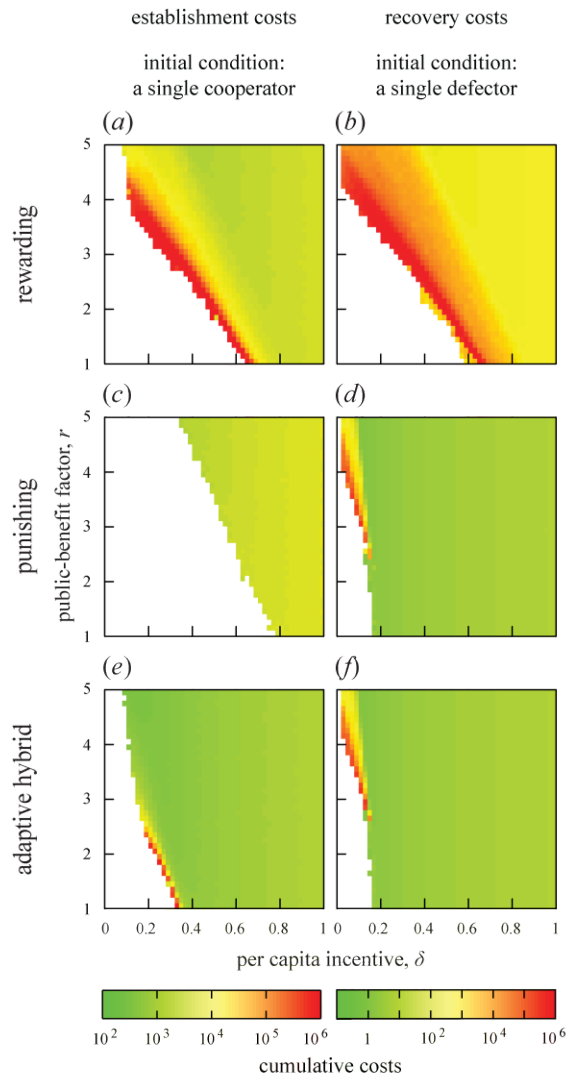

Figure S6-2. Costs for establishing and recovering full cooperation in spatial public good games with smaller population size. The adaptive hybrid policy is the least expensive also for populations on a 10×10 periodic square lattice. Parameters that are not varied are the same as in figure 2.

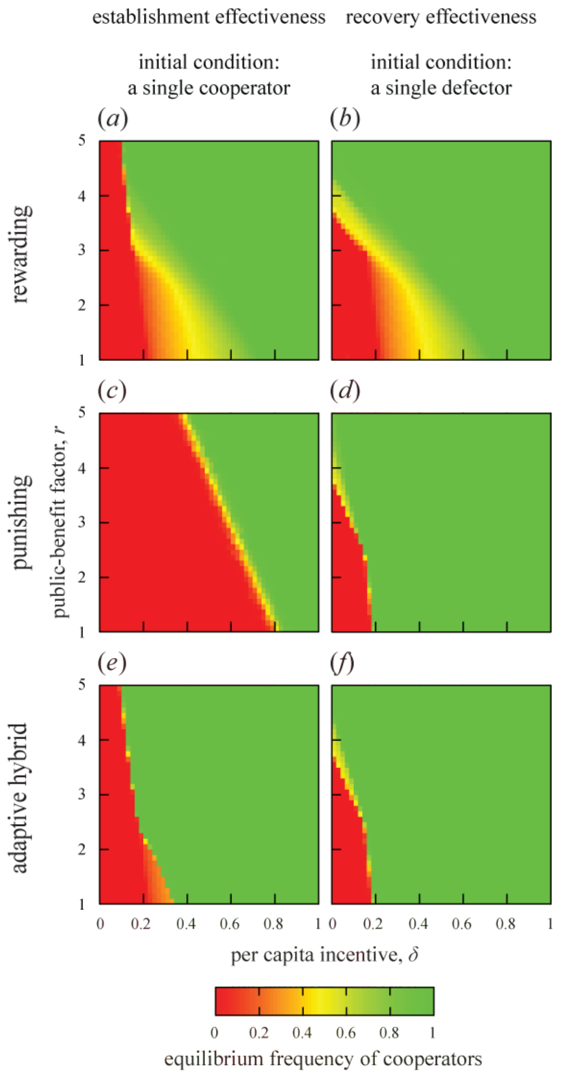

Figure S7-1. Effects of institutional sanctioning policies on spatial public good games with asynchronous updating. The adaptive hybrid policy has the broadest domain of success also when individual strategies are sequentially reviewed and updated. Parameters that are not varied are the same as in figure 2.

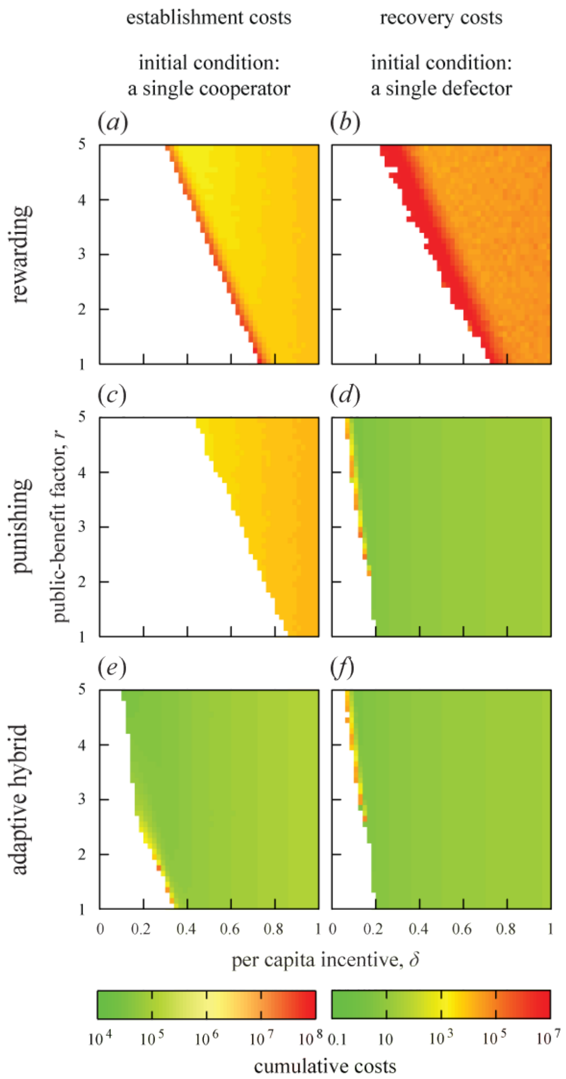

Figure S7-2. Costs for establishing and recovering full cooperation in spatial public good games with asynchronous updating. The adaptive hybrid policy is the least expensive also when individual strategies are sequentially reviewed and updated. Parameters that are not varied are the same as in figure 2.

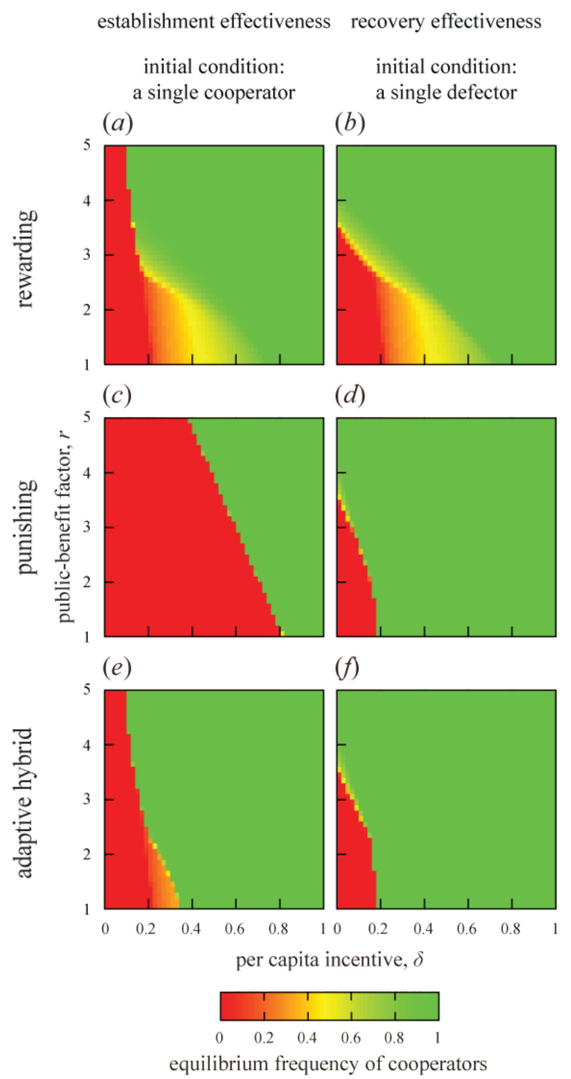

Figure S8-1. Effects of institutional sanctioning policies on spatial public good games with the proportional imitation rule. The adaptive hybrid policy has the broadest domain of success also when decisions on updating an individual's strategy follow the proportional imitation rule, in which the scaling factor Δ in equation (S1) is given by $n[c(r+1)+2n\delta]$. Parameters that are not varied are the same as in figure 2.

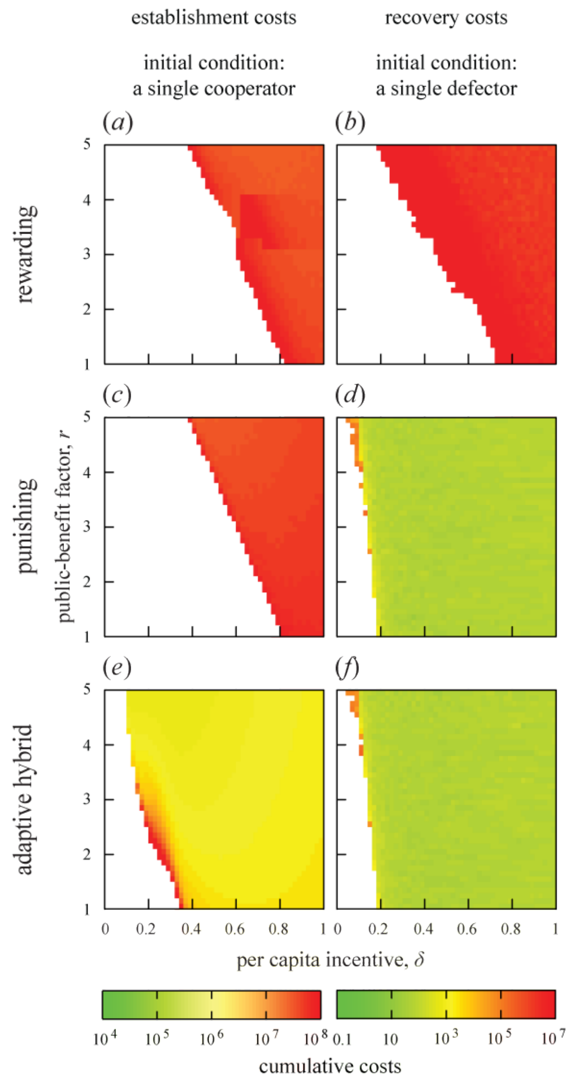

Figure S8-2. Costs for establishing and recovering full cooperation in spatial public good games with the proportional imitation rule. The adaptive hybrid policy is the least expensive also when decisions on updating an individual's strategy follow the proportional imitation rule. Parameters that are not varied are the same as in figure 2.

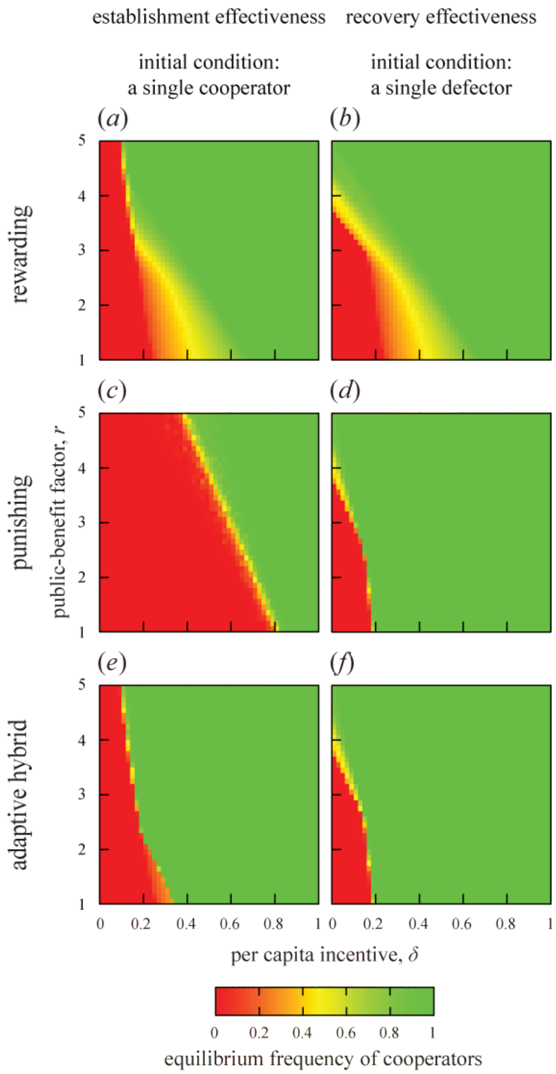

Figure S9-1. Effects of institutional sanctioning policies on spatial public good games with strategy-implementation errors. The adaptive hybrid policy has the broadest domain of success also when individuals participating in the public good game mistakenly use the strategy opposite to their own with probability $u_1 = 0.01$. Parameters that are not varied are the same as in figure 2.

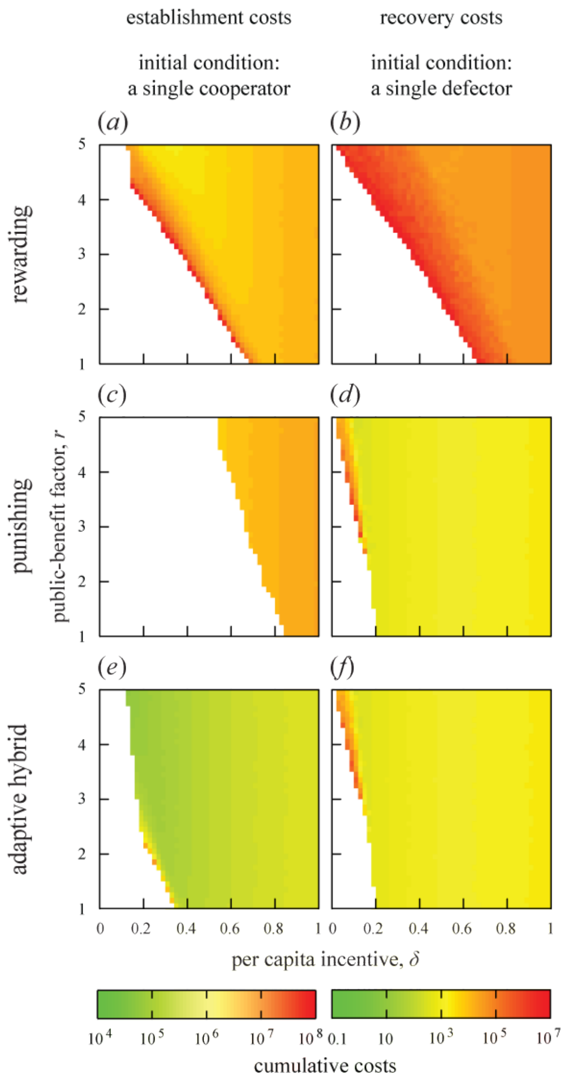

Figure S9-2. Costs for establishing and recovering full cooperation in spatial public good games with strategy-implementation errors. The adaptive hybrid policy is the least expensive also when individuals participating in the public good game mistakenly use the strategy opposite to their own with probability $u_1 = 0.01$. Parameters that are not varied are the same as in figure 2.

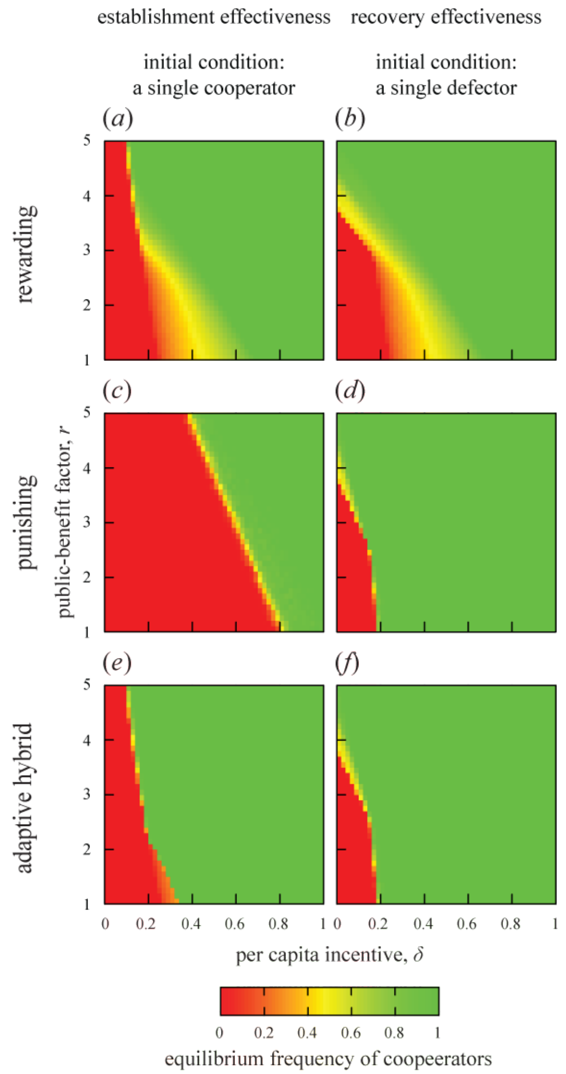

Figure S10-1. Effects of institutional sanctioning policies on spatial public good games with strategy-observation errors. The adaptive hybrid policy has the broadest domain of success also when the sanctioning institution mistakenly observes the opposite of an individual's strategy with probability $u_2 = 0.01$. Parameters that are not varied are the same as in figure 2.

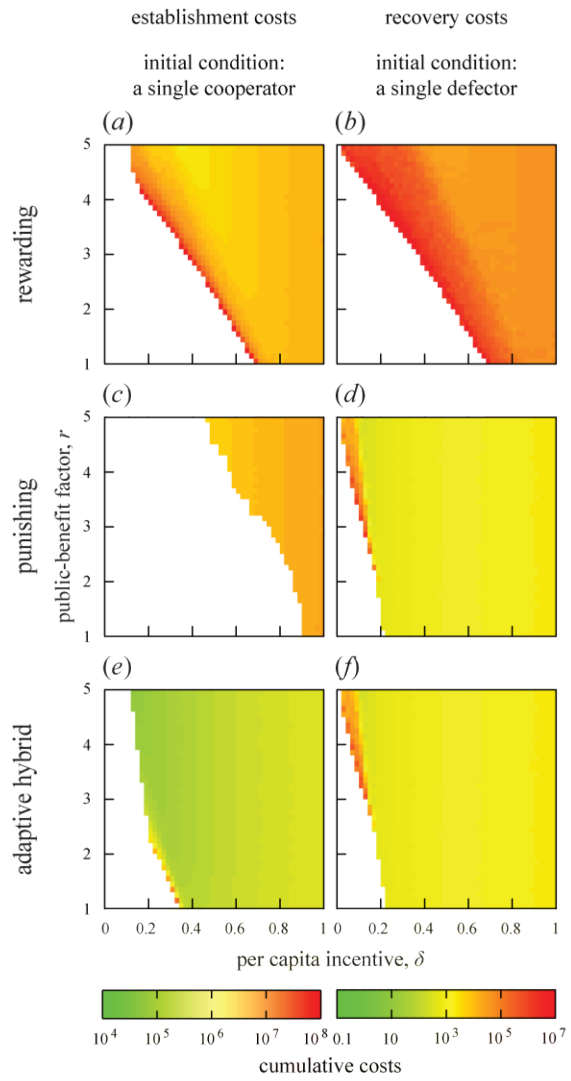

Figure S10-2. Costs for establishing and recovering full cooperation in spatial public good games with strategy-observation errors. The adaptive hybrid policy is the least expensive also when the sanctioning institution mistakenly observes the opposite of an individual's strategy with probability $u_2 = 0.01$. Parameters that are not varied are the same as in figure 2.

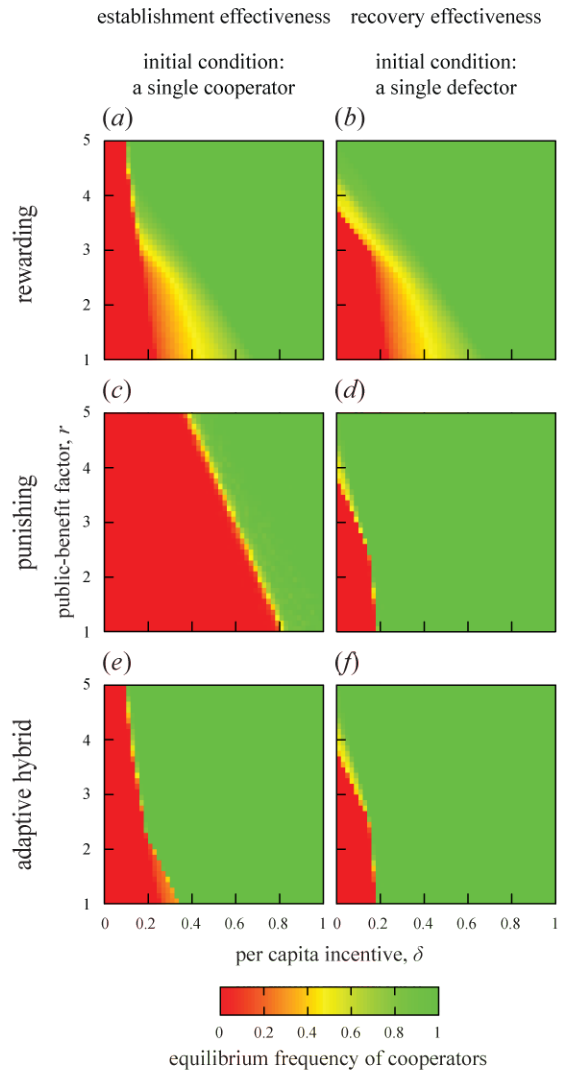

Figure S11-1. Effects of institutional sanctioning policies on spatial public good games with incentive-distribution errors. The adaptive hybrid policy has the broadest domain of success also when the institution, in the course of distributing incentives to individuals in an interaction group, mistakenly applies an incentive intended for an individual to another individual randomly selected from the group with probability $u_3 = 0.01$. Parameters that are not varied are the same as in figure 2.

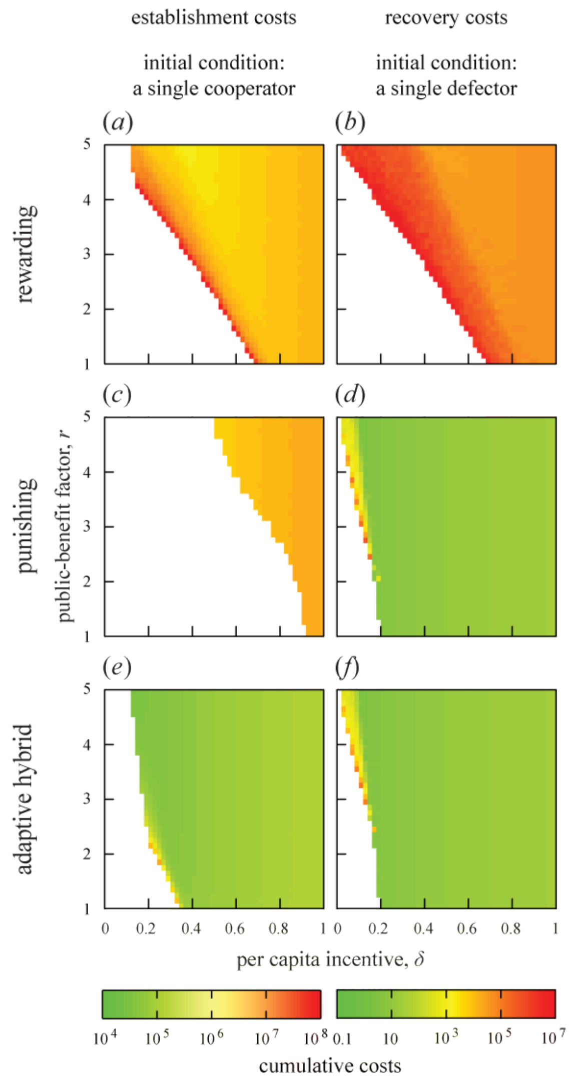

Figure S11-2. Costs for establishing and recovering full cooperation policies in spatial public good games with incentive-distribution errors. The adaptive hybrid policy is the least expensive also when the institution, in the course of distributing incentives to individuals in an interaction group, mistakenly applies an incentive intended for an individual to another individual randomly selected from the group with probability $u_3 = 0.01$. Parameters that are not varied are the same as in figure 2.

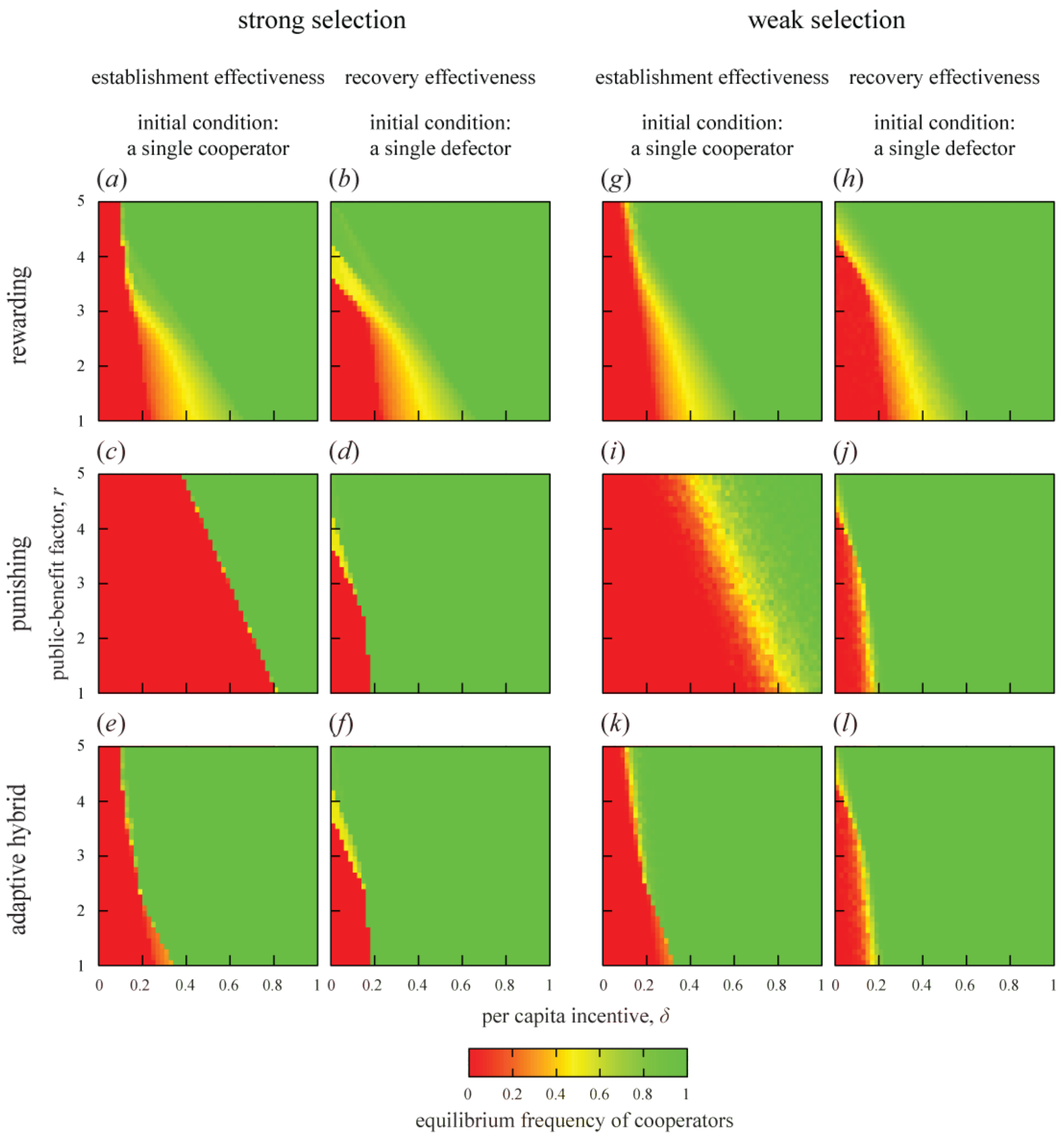

Figure S12-1. Effects of institutional sanctioning policies on spatial public good games with different payoff-observation errors. The adaptive hybrid policy has the broadest domain of success also when the ability of individuals to correctly update their strategy is varied. The Fermi function in the individual updating rule (§S1) is considered for two different values of the strength s of selection. The two columns on the left and on the right, respectively, correspond to stronger selection ($s = 100$), and thus less payoff-observation errors, and to weaker selection ($s = 1$), and thus more payoff-observation errors, than in our main model ($s = 10$ in figure 2). Parameters that are not varied are the same as in figure 2.

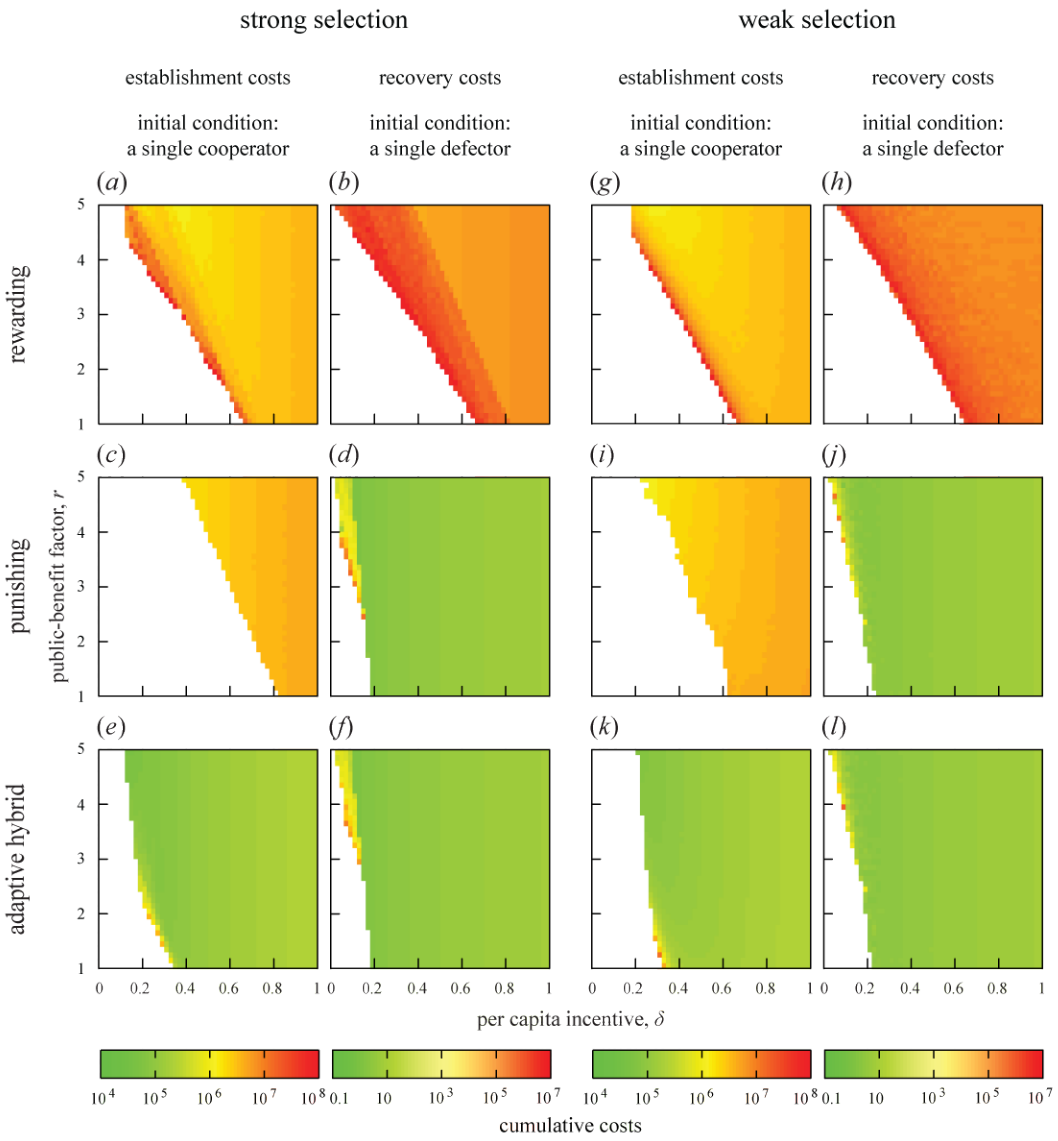

Figure S12-2. Costs for establishing and recovering full cooperation policies in spatial public good games with varying payoff-observation errors. The adaptive hybrid policy is the least expensive also when the ability of individuals to correctly update their strategy is varied. The Fermi function in the individual updating rule (§S1) is considered for varying degree of the strength s of selection. The two columns on the left and on the right correspond, respectively, to stronger selection ($s = 100$), and thus less payoff-observation errors, and to weaker selection ($s = 1$), and thus more payoff-observation errors, than in our main model ($s = 10$ in figure 2). Parameters that are not varied are the same as in figure 2.

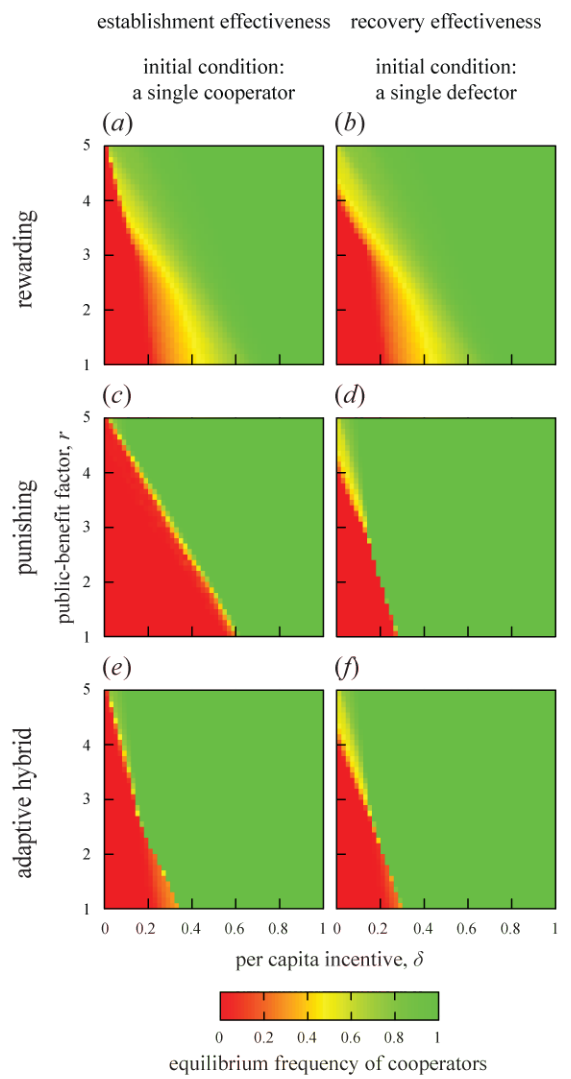

Figure S13-1. Effects of institutional sanctioning policies on spatial public good games with strategy-imitation errors. The adaptive hybrid policy has the broadest domain of success also if, when imitating a neighbour's strategy, the focal individual mistakenly chooses the opposite strategy with probability $u_5 = 0.01$. Parameters that are not varied are the same as in figure 2.

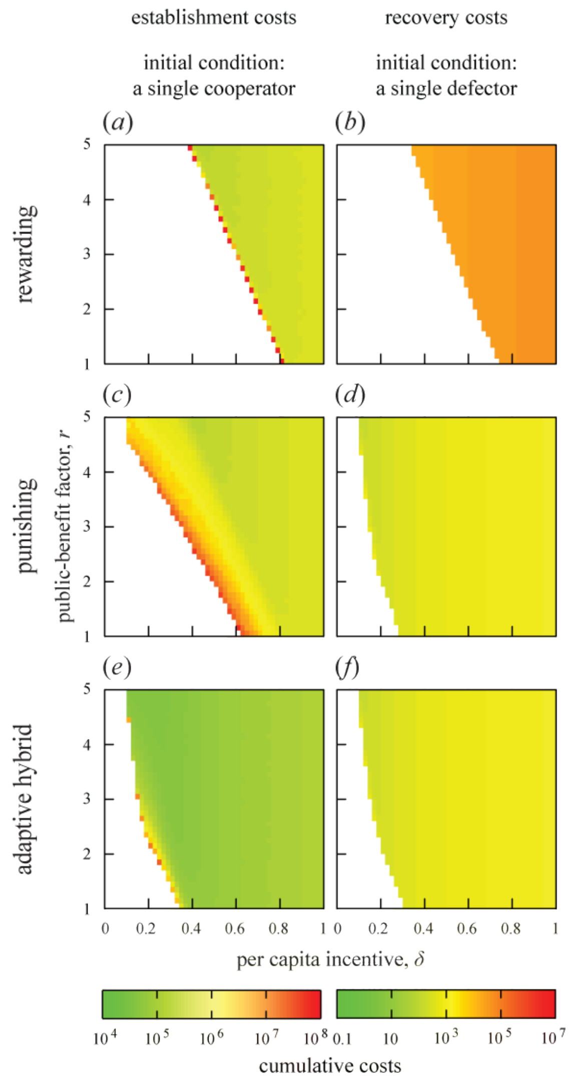

Figure S13-2. Costs for establishing and recovering full cooperation in spatial public good games with strategy-imitation errors. The adaptive hybrid policy is the least expensive also if, when imitating a neighbour's strategy, the focal individual mistakenly chooses the opposite strategy with probability $u_5 = 0.01$. As the equilibrium state of this model variant always includes some defectors, we consider cooperation to be established when the fraction of cooperators exceeds a threshold of 99%. For recovery costs, we consider the average cost over 1,000 time steps. The white regions in the right column show parameter combinations for which the fraction of cooperators fell below this threshold and did not recover within the given time frame. Results are qualitatively unchanged for other thresholds and time frames. Parameters that are not varied are the same as in figure 2.

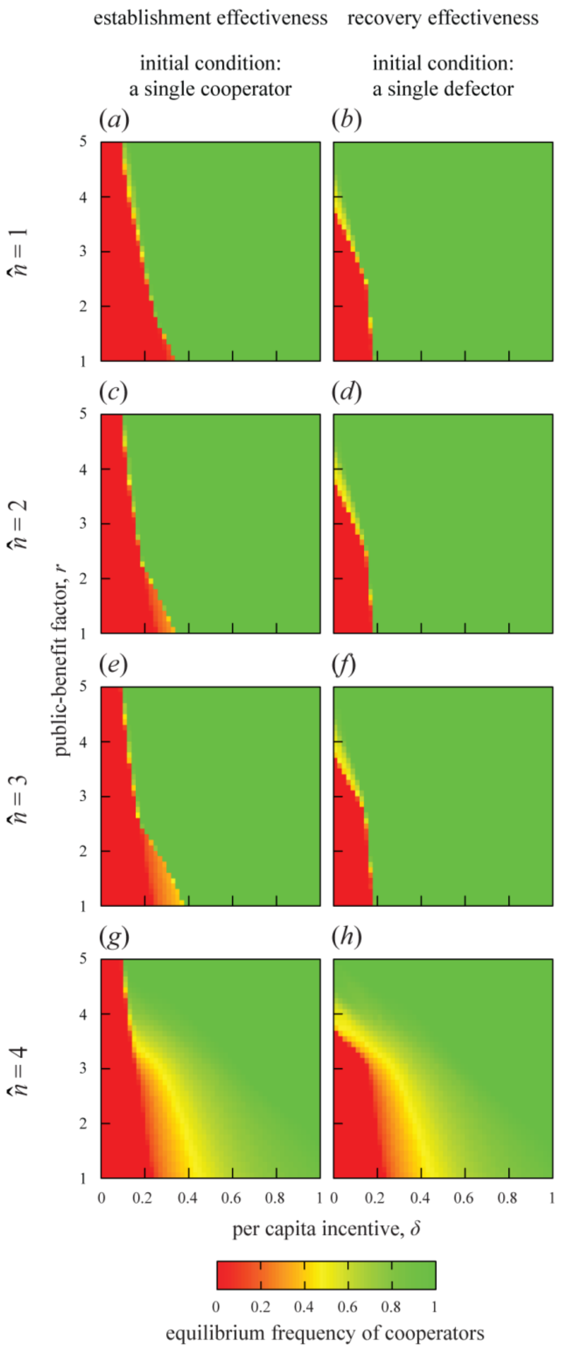

Figure S14-1. Effects on spatial public good games of institutional sanctioning policies based on on-off controls with varying switching thresholds. Switching from rewarding to punishing when the number of cooperators in a group exceeds $\hat{n} = 2$ is the most effective on-off control for establishing full cooperation. This can be compared with the theoretical prediction of optimal switching at 50% cooperators in well-mixed populations, implying $\hat{n} = 2.5$ for an interaction neighbourhood of five individuals ($n = 5$). Parameters that are not varied are the same as in figure 2.

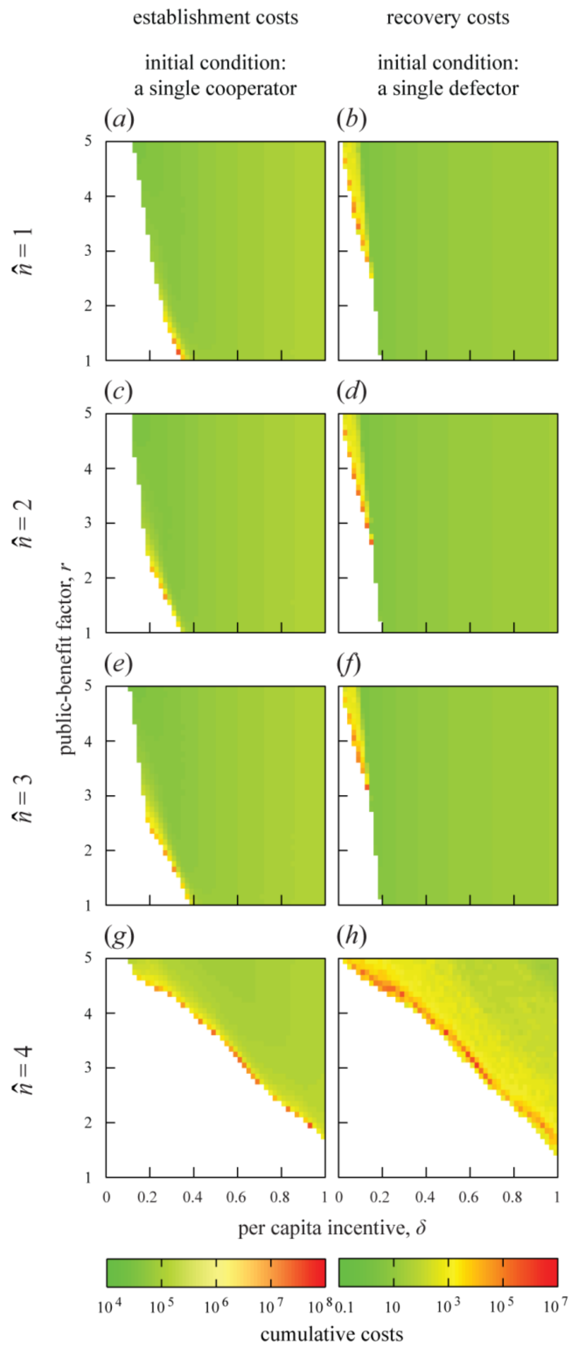

Figure S14-2. Costs for establishing and recovering full cooperation in spatial public good games for on-off controls with different switching thresholds. Switching from rewarding to punishing when the number of cooperators in a group exceeds $\hat{n} = 2$ is the least expensive on-off control for establishing full cooperation. Parameters that are not varied are the same as in figure 2.

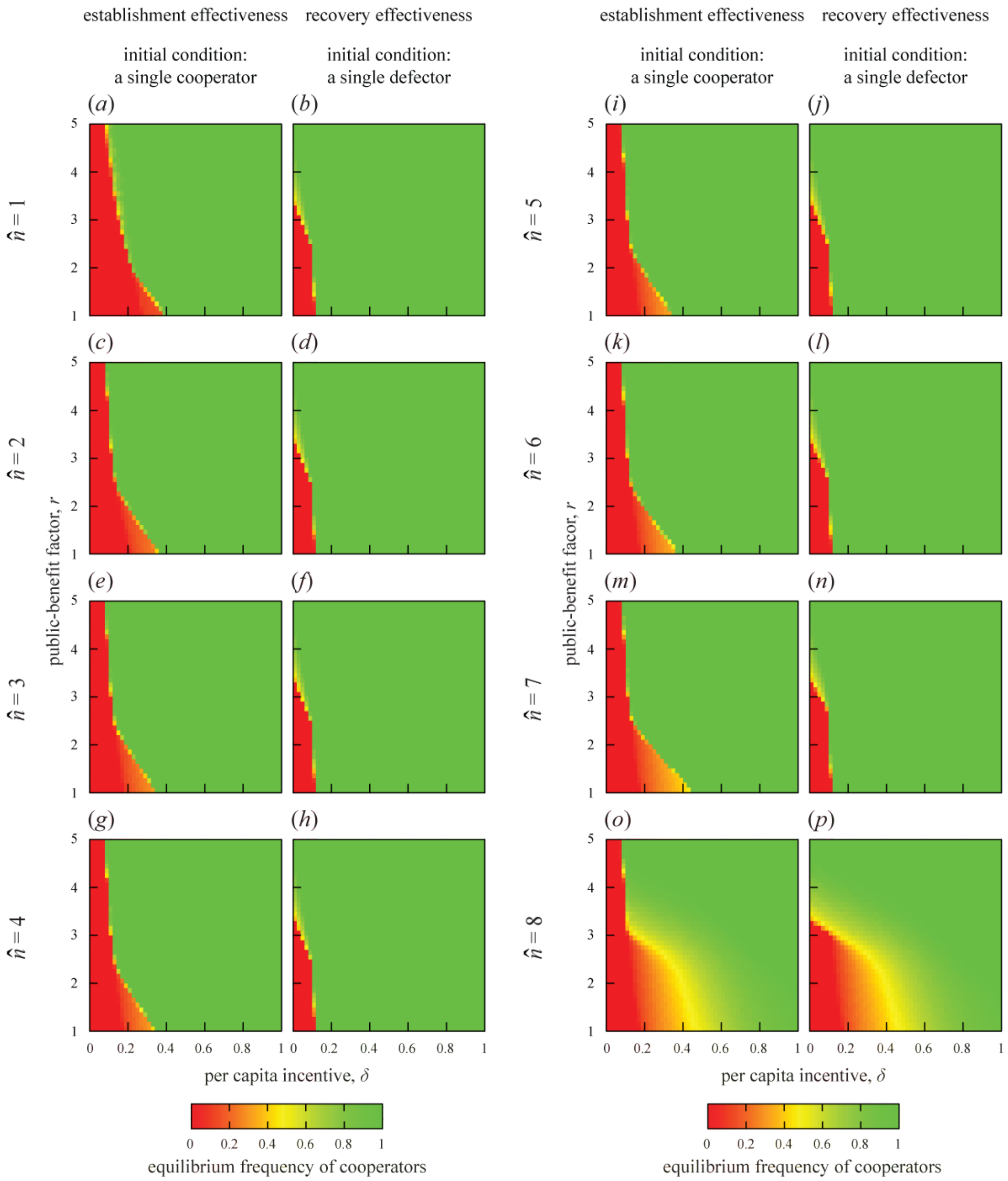

Figure S14-3. Effects on spatial public good games with an enlarged interaction neighbourhood of institutional sanctioning policies based on on-off controls with varying switching thresholds. Switching from rewarding to punishing when the number of cooperators in a group exceeds $\hat{n} = 4$ is the most effective on-off control for establishing full cooperation when the interaction neighbourhood consists of nine individuals ($n = 9$). This can be compared with the theoretical prediction of optimal switching at 50% cooperators in well-mixed populations, implying $\hat{n} = 4.5$ for an interaction neighbourhood of nine individuals. Parameters that are not varied are the same as in figure 2.

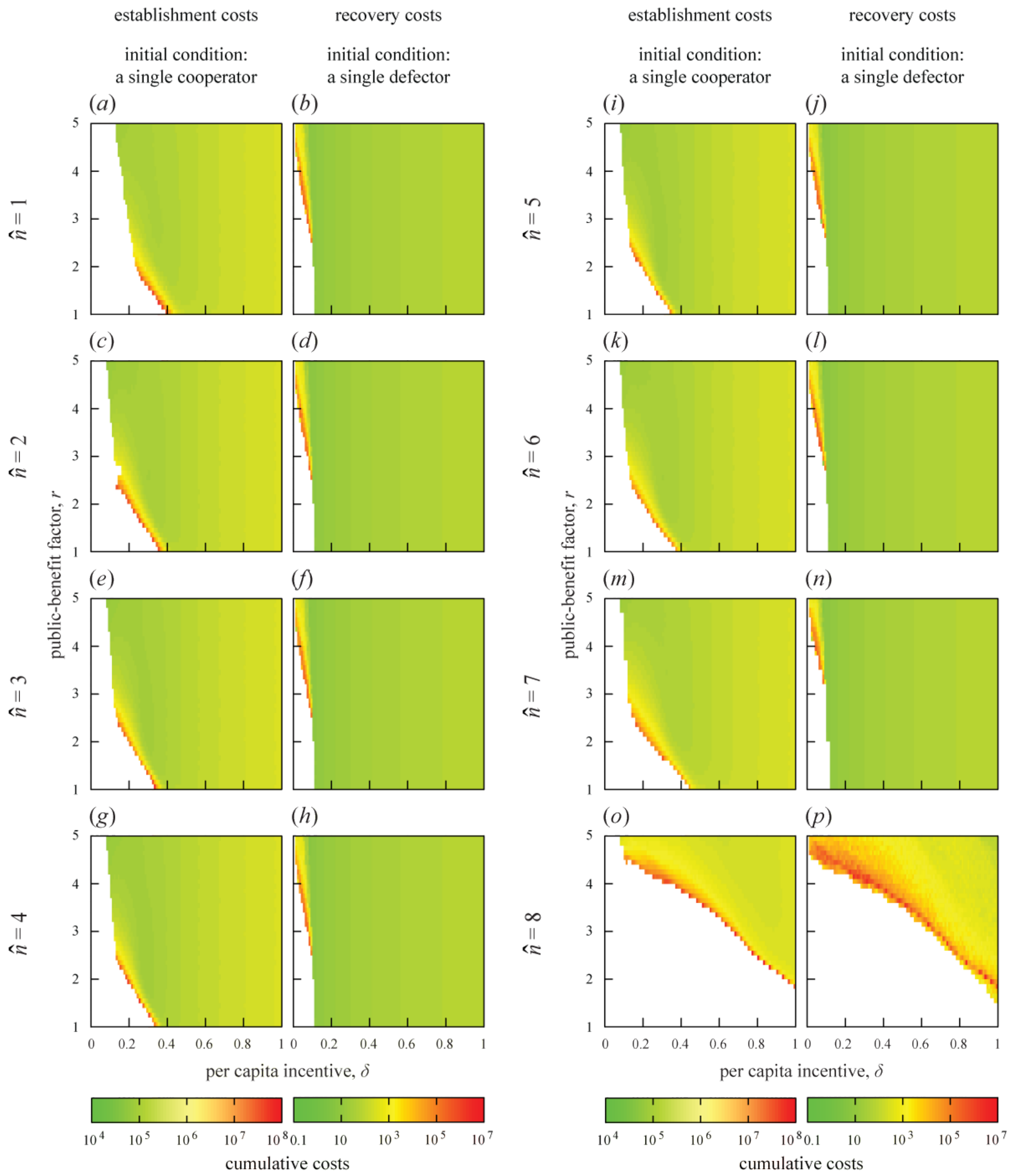

Figure S14-4. Costs for establishing and recovering full cooperation in spatial public good games with an enlarged interaction neighbourhood for on-off controls with different switching thresholds. Switching from rewarding to punishing when the number of cooperators in a group exceeds $\hat{n} = 4$ is the least expensive on-off control for establishing full cooperation when the interaction neighbourhood consists of nine individuals ($n = 9$). Parameters that are not varied are the same as in figure 2.
